# Postoperative morbidity and quality of life between totally laparoscopic total gastrectomy and laparoscopy-assisted total gastrectomy: a propensity-score matched analysis

**DOI:** 10.1186/s12885-021-08744-1

**Published:** 2021-09-11

**Authors:** Shin-Hoo Park, Yun-Suhk Suh, Tae-Han Kim, Yoon-Hee Choi, Jong-Ho Choi, Seong-Ho Kong, Do Joong Park, Hyuk-Joon Lee, Han-Kwang Yang

**Affiliations:** 1 Department of Surgery, Seoul National University College of Medicine, 101 Daehark-ro, Jongno-gu Seoul, 03080 Korea; 2 Department of Surgery, Seoul National University Hospital, 103 Daehark-ro, Jongno-gu Seoul, 03080 Korea; 3 Department of Foregut Surgery, Korea University Anam Hospital, Seoul, South Korea; 4 Department of Surgery, Seoul National University Bundang Hospital, 137-82 Gumiro, Bundang-gu, Seongnam-si, Gyeonggi-do 13620 South Korea; 5 Department of Surgery, Gyeongsang National University Changwon Hospital, Changwon, South Korea; 6 Division of Medical Statistics, Medical Research Collaborating Center, Seoul National University Hospital, Seoul, South Korea; 7 Cancer Research Institute, Seoul National University College of Medicine, Seoul, South Korea

**Keywords:** Gastric cancer, Totally laparoscopic total gastrectomy, Laparoscopy-assisted total gastrectomy, Morbidity, Quality of life, Hemi-double stapling

## Abstract

**Background:**

This study aimed to evaluate the surgical outcome and quality of life (QoL) of totally laparoscopic total gastrectomy (TLTG) compared with laparoscopy-assisted total gastrectomy (LATG) in patients with clinical stage I gastric cancer.

**Methods:**

From 2012 to 2018, EGC patients who underwent TLTG (*n* = 223), including the first case with intracorporeal hemi-double stapling, were matched to those who underwent LATG (*n* = 114) with extracorporeal circular stapling, using 2:1 propensity score matching (PSM). Prospectively collected morbidity was compared between the TLTG and LATG groups in conjunction with the learning curve. The European Organization for Research and Treatment of Cancer (EORTC) QoL questionnaires QLQ-C30, STO22, and OG25 were prospectively surveyed during postoperative 1 year for patient subgroups.

**Results:**

After PSM, grade I pulmonary complication rate was lower in the TLTG group (*n* = 213) than in the LATG group (*n* = 111) (0.5% vs. 5.4%, *P* = 0.007). Other complications were not different between the groups. The learning curve of TLTG was overcome at the 26th case in terms of the comprehensive complication index. The TLTG group after learning curve showed lower grade I pulmonary complication rate than the matched LATG group (0.5% vs. 4.7%, *P =* 0.024). Regarding postoperative QoL, the TLTG group (*n* = 63) revealed less dysphagia (*P* = 0.028), pain (*P* = 0.028), eating restriction (*P* = 0.006), eating (*P* = 0.004), odynophagia (*P =* 0.023) than the LATG group (*n* = 21). Multivariate analyses for each QoL item demonstrated that TLTG was the only common independent factor for better QoL.

**Conclusions:**

TLTG reduced grade I pulmonary complications and provided better QoL in dysphagia, pain, eating, odynophagia than LATG for patients with clinical stage I gastric cancer.

**Supplementary Information:**

The online version contains supplementary material available at 10.1186/s12885-021-08744-1.

## Introduction

The Korean national cancer screening program contributed to an increase in the diagnosis of early gastric cancer (EGC), reaching 61% in 2014. In particular, the incidence of upper one-third EGC gradually increased from 11.2% in 1995 to 16.0% in 2014, according to the A Information Committee of Korean Gastric Cancer [1, 2] The global incidence of cardia cancer has also grown seven-fold over the past decades [3]. In the era of minimally invasive surgery, laparoscopy-assisted total gastrectomy (LATG) or totally laparoscopic total gastrectomy (TLTG) have been highlighted with the expectation of minimal invasiveness. TLTG has not been fully standardized yet due to the technical difficulty of intracorporeal esophagojejunostomy. Even a recent large prospective multicenter phase II trial (KLASS-03) reported acceptable postoperative morbidity and mortality for patients with clinical stage I gastric cancer, the procedures for esophagojejunostomies were not standardized yet [4].

Previous studies using those various surgical procedures reported the potential advantages of TLTG, such as less pain, less blood loss, and shorter operation time, than LATG [5–10]. Besides, upper abdominal pain interferes with diaphragmatic movement and subsequently worsens pulmonary complications, which can be the typical morbidity after TLTG or LATG [11–13]. However, level I evidence for these morbidities has not been established by a randomized critical trial yet, and a reasonable case matching study with a sufficient sample size also has not even been reported. On the other hand, evaluating the postoperative quality of life (QoL) may provide meaningful implications for minimally invasive surgery. Less adhesion after laparoscopic gastrointestinal surgery was reported to improve QoL such as global health status, reflux symptom, and appetite loss by enabling a comfortable diet with less pain and better peristalsis [14–17]. However, QoL after TLTG was rarely compared with that after LATG, especially at multiple time points after surgery [18].

This study aimed to evaluate and compare surgical outcomes and QoL of TLTG with those of LATG in patients with clinical stage I gastric cancer using propensity-score matching (PSM).

## Materials and methods

### Study design

We reviewed the prospectively collected morbidity database of consecutive patients who underwent TLTG for clinical stage I gastric cancer between 2012 and 2018 at Seoul National University Hospital (SNUH). Clinical staging was evaluated by preoperative esophagogastroduodenoscopy, endoscopic ultrasonography and computed tomography. In this study, TLTG was defined as the case in which esophagojejunostomy was reconstructed intracorporeally, irrespective of intra- or extracorporeal jejunostomy. In SNUH, TLTG was performed since 2013, and four surgeons have gradually adopted TLTG according to the trend of minimal invasiveness demand, rather than separate indications for TLTG or LATG (Supplementary Fig. S1). All TLTG cases, including the first starting case with intracorporeal esophagojejunostomy by using the hemi-double stapling technique (hDST) were enrolled in this study to elucidate the safe adoption of new laparoscopic surgical skill and minimize the selection bias. TLTGs with intracorporeal esophagojejunostomy other than hDST or reduced port laparoscopic total gastrectomies were excluded (Supplementary Fig. S2).

Clinicopathologic data and other operative parameters were retrospectively reviewed. Each case from the TLTG group was 2:1 propensity score matched to control cases of the LATG group. The matching variables included age, sex, body mass index (BMI), combined organ resection, and pathological T and N stages. A propensity score of each patient was estimated by logistic regression (SPSS version 25; IBM Inc., Chicago, IL, USA) and matched nearest-neighbor value within a caliper 0.02 times the standard deviation of the estimated score. After PSM, the balance of covariates between TLTG and LATG group were evaluated by calculating the standardized mean difference. Detailed method for statistical analysis was described in supplementary methods.

### Surgical procedures

Laparoscopic total gastrectomy was conducted with D1+ lymph node dissection according to the Korean practice guideline for gastric cancer and Japanese gastric cancer guidelines [19, 20].

**Fig. 1:**
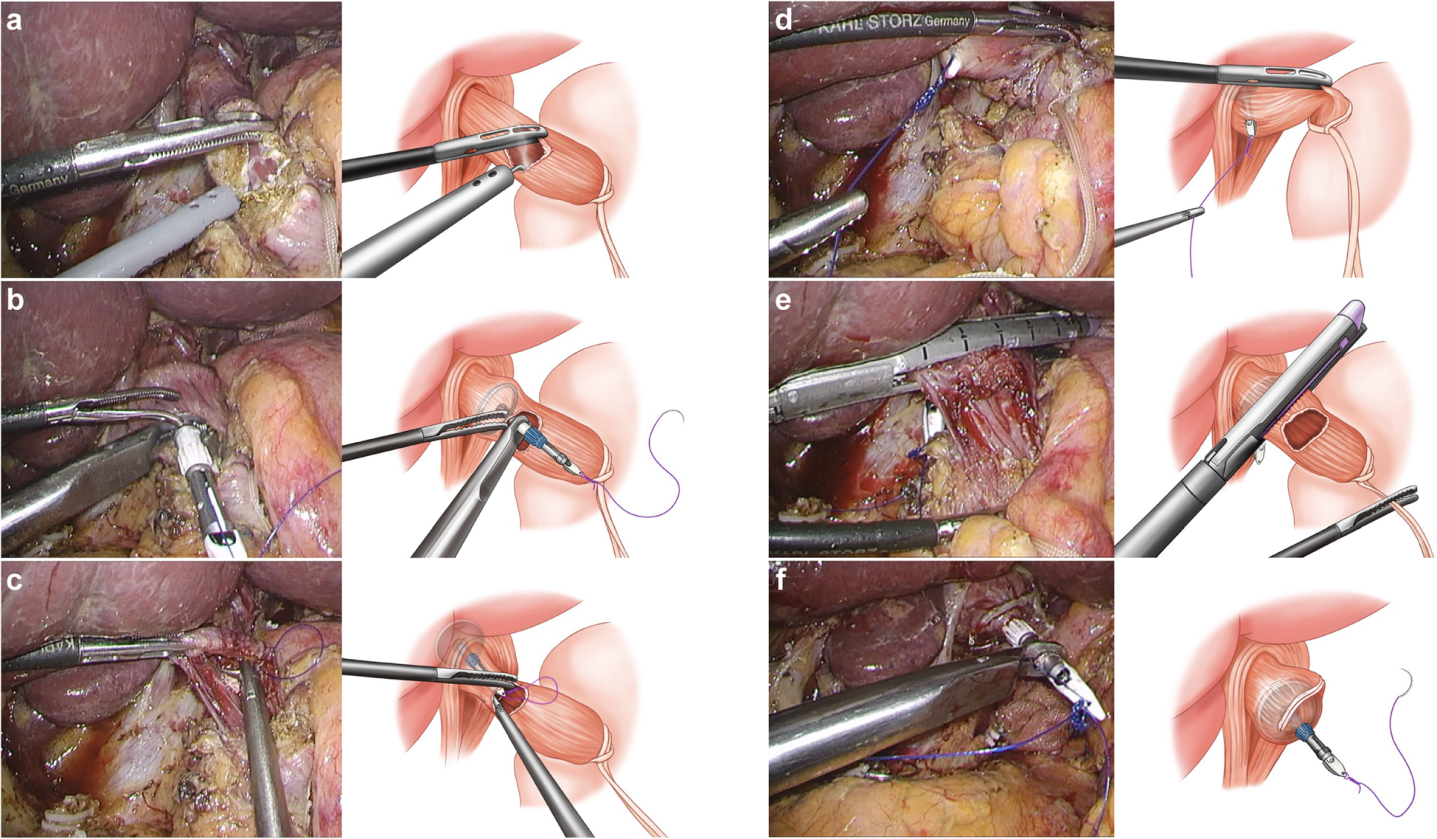
The hemi-double stapling technique for esophagojejunostomy in totally laparoscopic total gastrectomy. **a**. Opening of the anterior esophageal wall. **b**. The prepared anvil with stay suture was inserted through the distal esophagotomy site. **c**. The needle tip was pierced through the medial side of the esophagus. **d**. The anvil rod could be delivered outside by pulling the tied prolene. **e**. The distal esophagus was transected by linear stapler above the level of the esophagotomy site. **f**. The anvil shaft was positioned at the tip of stapling line to form hemi-double stapling

For TLTG, a 3–4 cm laparotomy was made in the umbilicus or through the left lower port site after transecting the duodenum. The anvil head of the circular stapler (EEA, 25–4.8 mm, Covidien, Mansfield, MA, USA), with its rod knotted several times using a 2–0 Prolene, were brought into the peritoneal cavity. The distal esophagus was fastened tightly with umbilical tape (32 mm width, 15 cm length, Ethicon, USA) and stretched in the direction of left lower quadrant. Then, the anterior wall of the distal esophagus was opened along the circumferential direction. The prepared anvil was inserted through the esophagotomy site and advanced into the esophagus higher than the expected proximal resection margin. By piercing the needle through the medial side of the esophageal wall, a spike of the anvil rod could be retrieved outside. The esophagus was transected by the linear stapler with 60-mm AMT, a purple cartridge (Endo GIA™, Covidien, Mansfield, MA, USA) above the esophagotomy site. As a result, the anvil rod is located at the medial end of the staple line (Fig. 1).

After the resected stomach was brought out through the mini-laparotomy, side-to side jejuno-jejunostomy was performed at approximately 40 cm distal to the expected esophagojejunostomy site using the linear stapler with 45- or 60-mm AVM, tan cartridge (Endo GIATM, Covidien). The circular stapler was inserted into the jejunal Roux limb, fastened with a rubber band to prevent slippage. Then, the Roux limb with the circular stapler was brought into the abdominal cavity, and pneumoperitoneum was reestablished. Under a secure laparoscopic view, the jejunal Roux limb was connected to the anvil, and intracorporeal anastomosis was finally performed. The jejunal stump was closed by the linear stapler with 60-mm AVM, a tan cartridge.

For LATG, about 8.5 cm sized upper midline incision was made at the epigastrium [21]. Under the direct vision through the mini-laparotomy, a purse-string suture and device were applied to the distal esophagus, and the stomach was transected distal to the purse-string device. The anvil head of the circular stapler (EEA, 25–4.8 mm, Covidien, Mansfield, MA, USA) was inserted into the esophagus and the purse-string suture was secured to fasten the anvil rod. Then, the extracorporeal esophagojejunsotomy was performed with a 25 mm circular stapler through a mini-laparotomy incision. Extracorporeal side to side jejuno-jejunostomy was performed through the mini-laparotomy incision with a similar manner to jejuno-jejunostomy in TLTG.

### Surgical outcome and quality of life

Complication data have been prospectively collected and recorded with the consensus of the entire gastrointestinal surgical team of SNUH through the weekly conference. General postoperative management including oral care, usage of prophylactic antibiotics, and pulmonary rehabilitation was the same over the study period. Morbidity and mortality were evaluated according to the Clavien-Dindo classification, and comprehensive complication index (CCI) calculated by the CCI formula (https://www.assessurgery.com/) [22]. Detailed methods for determining the learning curve based on cumulative sum score and evaluating the quality of life were described in supplementary methods.

## Results

### Surgical outcome

Before matching, the TLTG group (*n* = 223) and LATG group (*n* = 114) had no significant differences in baseline clinicopathologic variables (Table 1). After 2:1 PSM, the 213 patients in the TLTG group were matched to the 111 patients in the LATG group. The propensity scores, matching variables, and other remaining variables became highly balanced between TLTG and LATG groups (Supplementary Fig. S3). In terms of oncological safety, the number of retrieved lymph nodes and the distribution of TNM stage was not significantly different between TLTG and LATG (Table 1). In addition, the number of retrieved lymph nodes per each station was not significantly different across all stations between TLTG and LATG (Supplementary Fig. S4).

**Table 1:** Clinicopathologic characteristics between the TLTG group and LATG group before and after 2:1 PSM

Variables	Before matching		*P* value	After matching		*P* value
	TLTG (*n* = 223)	LATG (*n* = 114)		TLTG (*n* = 213)	LATG (*n* = 111)	
Age (years)	61.6 ± 11.1	59.5 ± 11.0	0.090	61.4 ± 10.7	59.8 ± 10.7	0.203
Sex			0.200			0.297
Male	166 (74.4)	77 (67.5)		158 (74.2)	76 (68.5)	
Female	57 (25.6)	37 (32.5)		55 (25.8)	35 (31.5)	
Body mass index (kg/m^2^)	24.2 ± 2.9	24.1 ± 3.3	0.960	24.2 ± 2.9	24.1 ± 3.3	0.665
Underlying disease						
Cerebrovascular disease	11 (4.9)	8 (7.0)	0.459	10 (4.7)	8 (7.2)	0.444
Dementia	1 (0.4)	0 (0)	0.474	1 (0.5)	0 (0)	0.470
Congestive (Ischemic) heart disease	20 (9.0)	4 (3.5)	0.075	19 (8.9)	4 (3.6)	0.109
Peripheral vascular disease	1 (0.4)	1 (0.9)	0.628	0 (0)	1 (0.9)	0.343
Hypertension	87 (39.0)	41 (36.0)	0.636	81 (38.0)	41 (36.9)	0.904
Pulmonary disease	13 (5.8)	4 (3.5)	0.439	13 (6.1)	4 (3.6)	0.436
Diabetes	42 (18.8)	16 (14.0)	0.290	41 (19.2)	16 (14.4)	0.356
Liver disease	10 (4.5)	1 (0.9)	0.107	7 (3.3)	0 (0)	0.100
Renal disease	5 (2.2)	1 (0.9)	0.668	5 (2.3)	0 (0)	0.169
Hemi- or paraplesia	1 (0.4)	0 (0)	0.474	1 (0.5)	0 (0)	0.470
Rheumatologic disorder	2 (0.9)	0 (0)	0.551	2 (0.9)	0 (0)	0.548
Any malignancies	4 (1.8)	2 (1.8)	0.979	3 (1.4)	1 (0.9)	0.695
Charlson comorbidity index						
Median [range]	1.0 [0–5]	0 [0–3]	0.104	1.0 [0–5]	0 [0–3]	0.118
0	99 (44.4)	60 (52.6)	0.259	95 (44.6)	58 (52.3)	0.266
1–2	107 (48.0)	49 (43.0)		103 (48.4)	49 (44.1)	
≥ 3	17 (7.6)	5 (4.4)		15 (7.0)	4 (3.6)	
Combined resection			0.295			0.574
None	195 (87.4)	106 (93.0)		192 (90.1)	103 (92.8)	
Gallbladder	25 (11.2)	7 (6.1)		21 (9.4)	7 (6.3)	
Spleen	3 (1.3)	1 (0.9)		1 (0.5)	1 (0.9)	
Tumor size (mm)	3.4 ± 1.8	3.2 ± 1.9	0.497	3.3 ± 1.7	3.2 ± 1.9	0.595
Proximal resected margin (cm)	2.6 ± 2.2	2.7 ± 2.3	0.799	2.6 ± 2.2	2.7 ± 2.4	0.702
Mean retrieved lymph nodes	46.2 ± 18.7	47.2 ± 15.8	0.603	46.8 ± 18.6	47.2 ± 15.6	0.827
Number of metastatic lymph nodes	0.8 ± 2.3	0.6 ± 2.5	0.578	0.7 ± 2.2	0.6 ± 2.5	0.880
R0 resection	223 (100)	114 (100)	**–**	213 (100)	111 (100)	**–**
Number of patients transfused	2 (0.9)	1 (0.9)	0.985	1 (0.5)	1 (0.9)	0.638
Operation time (minutes)	267.3 ± 50.3	277.6 ± 60.5	0.118	266.1 ± 49.5	278.5 ± 61.0	0.066
Hospital stay	11.9 ± 8.0	10.9 ± 6.9	0.216	11.8 ± 6.7	10.8 ± 6.7	0.222
pT category			0.201			0.274
pT1	146 (65.5)	85 (74.6)		144 (67.6)	82 (73.9)	
pT2	44 (19.7)	15 (13.2)		41 (19.2)	15 (13.5)	
pT3	25 (11.2)	8 (7.0)		22 (10.3)	8 (7.2)	
pT4	8 (3.6)	6 (5.3)		6 (2.8)	6 (5.4)	
pN category			0.250			0.413
pN0	177 (79.4)	99 (86.8)		171 (81.2)	96 (86.5)	
pN1	22 (9.9)	6 (5.3)		20 (9.4)	6 (5.4)	
pN2	18 (8.1)	5 (4.4)		15 (7.0)	5 (4.5)	
pN3	6 (2.7)	4 (3.5)		5 (2.3)	4 (3.6)	
TNM stage^a^			0.497			0.741
Stage I	174 (78.0)	95 (83.3)		169 (79.3)	92 (82.9)	
Stage II	29 (13.0)	12 (10.5)		27 (12.7)	12 (10.8)	
Stage III	20 (9.0)	7 (6.1)		17 (8.0)	7 (6.3)	
Stage IV	0 (0)	0 (0)		0 (0)	0 (0)	
Adjuvant chemotherapy	36 (16.1)	17 (14.9)	0.875	34 (16.0)	17 (15.3)	0.879

^a^TNM stage according to AJCC, the 7th edition

Abbreviations: *TLTG* totally laparoscopic total gastrectomy; *LATG* laparoscopy-assisted total gastrectomy; *PSM* propensity score matching

Regarding surgical complications within postoperative 1 month, grade I pulmonary complications of the TLTG group were significantly lower than those of the LATG group before (0.9% vs. 5.3%, *P =* 0.020) and after matching (0.5% vs. 5.4%, *P =* 0.007). However, the overall rate of pulmonary complications was not different between two groups before (10.8% vs. 12.3%, *p* = 0.717) and after matching (9.4% vs. 12.6%, *p* = 0.445). The other complications, including anastomosis-related complications, were not significantly different between the two groups before or after matching. Regarding complications detected between the postoperative 1 month and 1 year, the incidence and detection date of delayed stenosis of esophagojejunostomy were not different between the two groups before and after matching (Table 2). Feeding jejunostomy was not conducted in all cases of both TLTG and LATG groups.

**Table 2:** Postoperative complications between the TLTG and LATG groups before and after 2:1 PSM

Variables		Before matching		*P* value	After matching		*P* value
		TLTG (*n* = 223)	LATG (*n* = 114)		TLTG (*n* = 213)	LATG (*n* = 111)	
Overall complication: n (%)		61 (27.4)	33 (28.9)	0.798	58 (26.3)	32 (28.8)	0.693
Major complication (≥ grade IIIa)		26 (11.7)	13 (11.4)	0.945	24 (11.3)	13 (11.7)	0.905
Comprehensive complication index (median, range)		7.1 (0–60.2)	6.3 (0–40.5)	0.967	6.9 (0–60.2)	6.3 (0–40.5)	0.811
Hospital stay		11.9 ± 8.0	10.9 ± 6.9	0.216	11.8 ± 6.7	10.8 ± 6.7	0.222
Complication detected within 1 month							
Grade I	Wound	0 (0)	2 (1.8)	0.114	0 (0)	2 (1.8)	0.117
	Fluid collection	3 (1.3)	1 (0.9)	0.707	3 (1.4)	1 (0.9)	0.695
	Luminal bleeding	0 (0)	1 (0.9)	0.338	0 (0)	1 (0.9)	0.343
	Intestinal motility disorder^a^	2 (0.9)	0 (0)	0.551	2 (0.9)	0 (0)	0.548
	Organ ischemic change^b^	1 (0.4)	0 (0)	0.474	1 (0.5)	0 (0)	0.470
	Pulmonary	2 (0.9)	6 (5.3)	0.020	1 (0.5)	6 (5.4)	0.007
	Other systemic	3 (1.3)	2 (1.8)	0.769	3 (1.4)	2 (1.8)	0.785
Grade II	Fluid collection	9 (4.0)	2 (1.8)	0.345	9 (4.2)	2 (1.8)	0.343
	Intra-peritoneal bleeding^c^	1 (0.4)	0 (0)	0.474	1 (0.5)	0 (0)	0.470
	Luminal bleeding	1 (0.4)	1 (0.9)	0.628	1 (0.5)	1 (0.9)	0.638
	Stenosis (EJ)	0 (0)	2 (1.8)	0.114	0 (0)	2 (1.8)	0.117
	Intestinal motility disorder^a^	2 (0.9)	1 (0.9)	0.985	2 (0.9)	1 (0.9)	0.973
	Anastomosis site leakage	4 (1.8)	2 (1.8)	0.979	4 (1.9)	1 (0.9)	0.664
	Other fistula	0 (0)	1 (0.9)	0.338	0 (0)	1 (0.9)	0.343
	Organ ischemic change^d^	1 (0.4)	0 (0)	0.474	1 (0.5)	0 (0)	0.470
	Pulmonary	12 (5.4)	4 (3.3)	0.592	10 (4.7)	4 (3.6)	0.778
	Other systemic	8 (3.6)	4 (3.5)	0.971	8 (3.8)	4 (3.6)	0.945
Grade IIIa	Wound	2 (0.9)	3 (2.6)	0.341	2 (0.9)	3 (2.7)	0.343
	fluid collection	12 (5.4)	4 (3.5)	0.592	11 (5.1)	4 (3.6)	0.592
	Intra-peritoneal bleeding^e^	1 (0.4)	0 (0)	0.474	1 (0.5)	0 (0)	0.470
	Stenosis	1 (0.4)	0 (0)	0.474	1 (0.5)	0 (0)	0.470
	Intestinal motility disorder^f^	1 (0.4)	0 (0)	0.474	1 (0.5)	0 (0)	0.470
	Anastomosis site leakage	5 (2.2)	1 (0.9)	0.668	5 (2.3)	1 (0.9)	0.668
	Pulmonary	6 (2.7)	2 (1.8)	0.722	5 (2.3)	2 (1.8)	0.749
	Other systemic	1 (0.4)	0 (0)	0.474	1 (0.5)	0 (0)	0.470
Grade IIIb	Wound	2 (0.9)	1 (0.9)	0.985	2 (0.9)	1 (0.9)	0.973
	Intestinal motility disorder^f^	0 (0)	1 (0.9)	0.338	0 (0)	1 (0.9)	0.343
Grade IVa	Intra-peritoneal bleeding^g^	1 (0.4)	0 (0)	0.474	1 (0.5)	0 (0)	0.470
	Pulmonary	2 (0.9)	0 (0)	0.551	1 (0.5)	0 (0)	0.470
Grade IVb	Intra-peritoneal bleeding^h^	1 (0.4)	0 (0)	0.474	1 (0.5)	0 (0)	0.470
Complication detected from 1 month to 1 year							
	Delayed EJ stenosis (total no.)	14 (6.3)	6 (5.3)	0.811	14 (6.6)	6 (5.4)	0.810
	Delayed EJ stenosis (≥grade IIIa)	7 (3.1)	5 (4.4)	0.549	7 (3.3)	5 (4.5)	0.553
	Detected period for (Days)	76.8 ± 22.0	74.2 ± 12.7	0.743	76.8 ± 22.0	74.2 ± 12.7	0.743

Abbreviations: *TLTG* totally laparoscopic total gastrectomy, *LATG* laparoscopy-assisted total gastrectomy, *PSM* propensity score matching, EJ  esophagojejunostomy

Foot notes: ^a^ = Ileus; ^b^ = Splenic infarct; ^c^ = liver bed -bed side bleeding; ^d^ = Mesenteric infarct; ^e^ = Inferior epigastric arterial bleeding; ^f^ = Intestinal obstruction; ^g^ = splenic arterial bleeding; ^h^ = Splenic arterial and right gastric arterial stump bleeding

### Learning curve for TLTG

The CUSUM graph using the CCI showed two negatively sloping curve during the observation period with the trend line (y = − 0.3118x + 55.602) (Supplementary Fig. S5a). In the first phase, the CUSUM score gradually increased and reached the first highest peak at case 26 (score, 118.00), then two more peak values at case 50 (score, 113.77) and case 73 (score, 80.77), and decreased until case 103 (score, − 112.357). The TLTG group did not show clear decreasing pattern in operation time over chronological cases (Supplementary Fig. S5b). We defined the 26th case as a point of overcoming the learning curve, and rationales for this was described in Supplementary Table 1.

Table 3 presents the postoperative morbidity between the late TLTG group after overcoming the learning curve and the re-matched LATG group since 2012 when LATG was actively performed. The overall rate of grade I complication (2.1% vs 8.5%, *P =* 0.016), especially pulmonary complication (0.5% vs. 4.7%, *P* = 0.024), was still significantly lower in the late TLTG group than in the LATG group after matching. The overall rate of pulmonary complications was not different between two late groups (9.0% vs. 11.3%, *P* = 0.546). Other complications, including anastomosis-related complications, were not different between the late TLTG and LATG groups before and after matching.

**Table 3:** Postoperative complications between the late TLTG after learning curve and LATG groups. Learning curve of TLTG was determined after the 26th case

Variables	Before matching		*P* value	After matching		*P* value
	late TLTG after learning curve (*n* = 197)	LATG (*n* = 114)		late TLTG after learning curve (*n* = 188)	LATG (*n* = 106)	
Overall complication: n (%)	48 (24.4)	33 (28.9)	0.422	44 (23.4)	30 (28.3)	0.401
Major complication (≥ grade IIIa)	23 (11.7)	13 (11.4)	0.942	22 (11.7)	12 (11.3)	0.922
Comprehensive complication index (median, range)	6.5 (0–60.2)	6.3 (0–40.5)	0.553	6.5 (0–60.2)	6.3 (0–40.5)	0.521
Hospital stays	11.5 ± 7.5	10.9 ± 6.9	0.461	11.1 ± 7.1	11.9 ± 10.1	0.422
Complication detected within 1 month						
Grade I	6 (3.0)	10 (8.8)	0.034	4 (2.1)	9 (8.5)	0.016
Wound	0 (0)	2 (1.8)	0.134	0 (0)	2 (1.9)	0.129
Fluid collection	0 (0)	1 (0.9)	0.367	0 (0)	1 (0.9)	0.361
Luminal bleeding	0 (0)	1 (0.9)	0.367	0 (0)	1 (0.9)	0.361
Intestinal motility disorder^a^	1 (0.5)	0 (0)	0.446	1 (0.5)	0 (0)	0.452
Organ ischemic change^b^	1 (0.5)	0 (0)	0.446	0 (0)	0 (0)	–
Pulmonary	2 (1.0)	6 (5.3)	0.055	1 (0.5)	5 (4.7)	0.024
Other systemic	1 (0.5)	2 (1.8)	0.557	1 (0.5)	2 (1.9)	0.296
Grade II	25 (12.7)	14 (12.3)	0.916	24 (12.8)	13 (12.3)	0.901
Fluid collection	6 (3.0)	2 (1.8)	0.715	6 (3.2)	2 (1.9)	0.715
Intra-peritoneal bleeding^c^	1 (0.5)	0 (0)	0.446	1 (0.5)	0 (0)	0.452
Luminal bleeding	0 (0)	1 (0.9)	0.367	0 (0)	1 (0.9)	0.361
Stenosis (EJ)	0 (0)	2 (1.8)	0.134	0 (0)	2 (1.9)	0.129
Intestinal motility disorder^a^	1 (0.5)	1 (0.9)	0.694	1 (0.5)	1 (0.9)	0.680
Anastomosis site leakage	3 (1.5)	2 (1.8)	0.876	3 (1.6)	2 (1.9)	0.853
Other fistulas	0 (0)	1 (0.9)	0.367	0 (0)	1 (0.9)	0.361
Organ ischemic change^d^	1 (0.5)	0 (0)	0.446	1 (0.5)	0 (0)	0.452
Pulmonary	9 (4.6)	4 (3.5)	0.774	9 (4.8)	3 (2.8)	0.547
Other systemic	7 (3.6)	4 (3.5)	0.984	7 (3.7)	4 (3.8)	0.983
Grade IIIa	22 (11.2)	10 (8.8)	0.565	21 (11.2)	9 (8.5)	0.550
Wound	2 (1.0)	3 (2.6)	0.360	2 (1.1)	2 (1.9)	0.621
fluid collection	11 (5.6)	4 (3.5)	0.585	10 (5.3)	4 (3.8)	0.777
Intra-peritoneal bleeding^e^	1 (0.5)	0 (0)	0.446	1 (0.5)	0 (0)	0.452
Stenosis	0 (0)	0 (0)	–	0 (0)	0 (0)	–
Intestinal motility disorder^f^	1 (0.5)	0 (0)	0.446	1 (0.5)	0 (0)	0.452
Anastomosis site leakage	5 (2.5)	1 (0.9)	0.420	5 (2.7)	1 (0.9)	0.424
Pulmonary	5 (2.5)	2 (1.8)	0.653	4 (2.1)	2 (1.9)	0.888
Other systemic	1 (0.5)	0 (0)	0.446	1 (0.5)	0 (0)	0.452
Grade IIIb	2 (1.0)	2 (1.8)	0.626	2 (1.1)	2 (1.9)	0.621
Wound	2 (1.0)	1 (0.9)	0.904	2 (1.1)	1 (0.9)	0.921
Intestinal motility disorder^f^	0 (0)	1 (0.9)	0.367	0 (0)	1 (0.9)	0.361
Grade IVa	2 (1.0)	0 (0)	0.534	2 (1.1)	0 (0)	0.537
Intra-peritoneal bleeding^g^	1 (0.5)	0 (0)	0.446	1 (0.5)	0 (0)	0.452
Pulmonary	1 (0.5)	0 (0)	0.446	1 (0.5)	0 (0)	0.452
Grade IVb	1 (0.5)	0 (0)	0.446	1 (0.5)	0 (0)	0.452
Intra-peritoneal bleeding^h^	1 (0.5)	0 (0)	0.446	1 (0.5)	0 (0)	0.452
Complication detected from 1 month to 1 year						
Delayed EJ stenosis (total no.)	13 (6.6)	6 (5.3)	0.807	12 (6.4)	5 (4.7)	0.615
Delayed EJ stenosis (≥grade IIIa)	7 (3.6)	5 (4.4)	0.764	6 (3.2)	4 (3.8)	0.751
Detected period for (Days)	73.6 ± 19.3	74.2 ± 12.7	0.942	77.4 ± 14.2	73.4 ± 14.0	0.607

Abbreviations: *TLTG* totally laparoscopic total gastrectomy; *LATG* laparoscopy-assisted total gastrectomy; *PSM* propensity score matching; *EJ* esophagojejunostomy

Foot notes: ^a^ = Ileus; ^b^ = Splenic infarct; ^c^ = liver bed -bed side bleeding; ^d^ = Mesenteric infarct; ^e^ = Inferior epigastric arterial bleeding; ^f^ = Intestinal obstruction; ^g^ = splenic arterial bleeding; ^h^ = Splenic arterial and right gastric arterial stump bleeding

### Quality of life

**Fig. 2:**
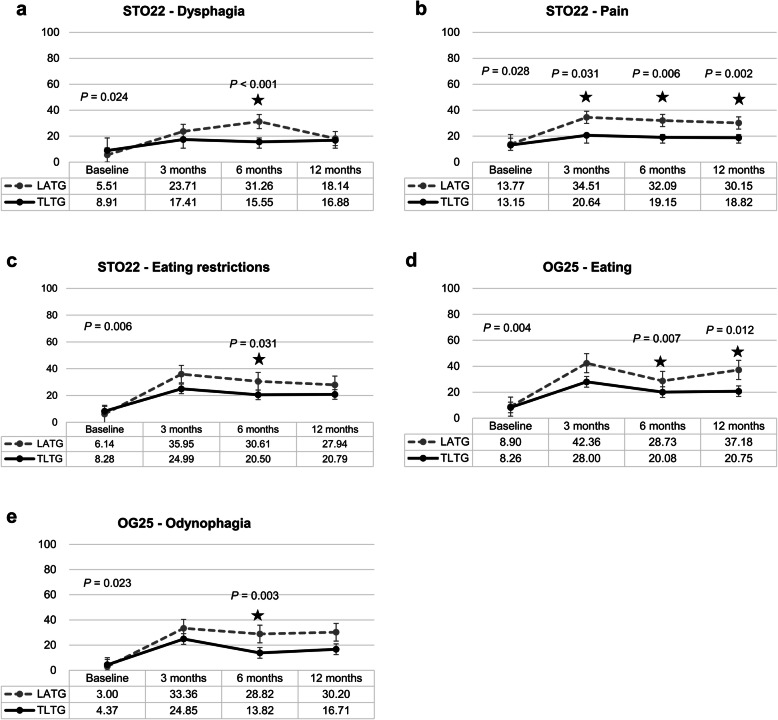
Quality of life between the TLTG group (*n* = 63) and LATG group (*n* = 21) using the EORTC. **a**. STO22: dysphagia. **b**. STO22: pain. **c**. STO 22: eating restriction. **d**. OG25: eating. **e**. OG25: odynophagia

The TLTG (*n* = 63) and LATG (*n* = 21) groups were matched to prospectively collected QoL data. The clinicopathologic characteristics and complications were not different between the two groups (Supplementary Table 2). During postoperative 1 year, the rates of STO22 dysphagia (*P =* 0.028), STO22 pain (*P =* 0.028), STO22 eating restriction (*P =* 0.006), OG25 eating (*P* = 0.004), and OG25 odynophagia (*P* = 0.023) were significantly lower in the TLTG group (*n* = 63) than in the LATG group (*n* = 21) (Fig. 2a-e). Other QoL items of EORTC-C30, STO22, and OG25 questionnaires between the TLTG and LATG groups are presented in Supplementary Fig. S6. For those five significant QoL questionnaires, ANCOVA at each three different time points (postoperative 3 months, 6 months, and 1 year) revealed that STO22 dysphagia at postoperative 6 months (15.55 vs. 31.26, *P <* 0.001), STO22 pain at postoperative 3, 6 and 12 months (20.64 vs. 34.51, *P =* 0.031; 19.15 vs. 32.09, *P =* 0.006; 18.82 vs. 30.15, *P =* 0.002), STO22 eating restriction at postoperative 6 months (20.50 vs. 30.61, *P =* 0.031), OG25 eating at postoperative 6 and 12 months (20.08 vs. 28.73, *P =* 0.007; 20.75 vs. 37.18, *P =* 0.012), and OG25 odynophagia at postoperative 6 months (13.82 vs. 28.82, *P =* 0.003) were significantly better in the TLTG group than in LATG group, after controlling the confounding effects of preoperative QoL. Multivariate linear regression for variables including age, sex, BMI, TLTG (vs. LATG), pT stage, pN stage, baseline QoL score, and the rate of overall complication (CCI) revealed that TLTG was the only common independent risk factor for significantly better QoL at each different time point, after excluding all possible confounding factors (all *P* < 0.05) (Table 4). For more robust validation of the role of TLTG, we used the anastomosis related complication and motility disorder as covariates for multivariate analysis, instead of CCI as overall complications. Still, TLTG remained as the only common independent risk factor for better QoL (Supplementary Table 3).

**Table 4:** Analysis for variables determining the differences of symptom scale between the TLTG and LATG group

Symptom scale	Variable factors	Unstandardized coefficient		*P* value
		B	Standard errors	
STO22 dysphagia (6 months)	TLTG (vs LATG)	−20.928	5.234	< 0.001
STO22 pain (3 months)	TLTG (vs LATG)	−11.635	5.108	0.031
	STO22 pain (preoperative)	0.419	0.202	0.047
STO22 pain (6 months)	TLTG (vs LATG)	−16.975	5.061	0.002
STO22 pain (12 months)	TLTG (vs LATG)	−16.170	4.762	0.002
STO22 eating restriction (6 months)	TLTG (vs LATG)	−11.407	4.983	0.031
	STO22 eating (preoperative)	0.657	0.295	0.035
OG25 eating (6 months)	TLTG (vs LATG)	−11.793	3.969	0.007
	OG25 eating (preoperative)	0.663	0.230	0.008
OG25 eating (12 months)	TLTG (vs LATG)	−13.351	5.913	0.033
OG25 odynophagia (6 months)	TLTG (vs LATG)	−17.318	5.114	0.002

Abbreviations: *TLTG* totally laparoscopic total gastrectomy; *LATG* laparoscopy-assisted total gastrectomy

## Discussion

This study successfully demonstrated the advantage of TLTG compared with matched LATG in terms of lower grade I pulmonary complication rate and better QoL of dysphagia, pain, or eating during postoperative 1 year. Retrospective studies cannot usually be sensitive enough to analyze parameters such as minor complications or changes in QoL and may provide false-negative or biased results. This study utilized prospectively collected complication data and QoL cohort, both of which had been recruited independently of the original purpose of this study. We believe that our study can provide less biased and more sensitive results than other unmatched retrospective studies.

The postoperative pulmonary complication was reported as one of the greatest risk factors for postoperative mortality in gastric cancer patients [12, 23, 24]. In addition, total gastrectomy was an independent risk factor for pulmonary complications following laparoscopic gastrectomy [13]. Previous meta-analysis comparing LATG with open TG reported that LATG was associated with a significant reduction in medical complications, but a contribution from respiratory complications was not significant [25]. Other retrospective study limitedly demonstrated the lower incidence of pulmonary complications in the LATG group than in the OTG group, only in patients aged over 65 [26]. On the other hand, previous studies comparing TLTG and LATG mainly focused on anastomotic complications, and rarely addressed issues with pulmonary complications [1–5]. Upper abdominal incision causes decreased pulmonary function more frequently than lower abdominal surgery [11, 27, 28]. The mini-laparotomy wounds of the LATG are inevitably larger and located closer to epigastrium than those of TLTG. In addition, the LATG group had a higher score of STO22 pain than the TLTG group (Fig. 2b). The larger incisions in the epigastrium and worse pain score may explain the limited movement of the diaphragm and deep breathing, followed by a decreased pulmonary function in the LATG group. In this study, underlying pulmonary disease was not significantly different between TLTG and LATG groups, and the number of high Charlson comorbidity index (≥3) was limited in both groups (Table 1), which could be one explanation of limited difference in low grade pulmonary complication only. Considering the possible risk of atelectasis to serious respiratory failure especially in underlying comorbid patients [29, 30], TLTG can be more meaningful in patients with underlying pulmonary disease.

This is the first study comparing QoL over consecutive multiple time points during the year after operation between TLTG and LATG groups. Previous studies reported better QoL scores of C30 pain and STO22 dysphagia in the TLTG group than in the LATG group, but only investigated the QoL at a single time point and did not include OG25, more sensitive in evaluating QoL after total gastrectomy [18, 31]. The changes of QoL associated with surgical procedures were mainly determined during the early recovery period [32]. Most acute changes of postoperative QoL gradually became stable during the first year following surgery [33, 34], and tend to be recovered close to preoperative QoL in about 1 year after gastrectomy [33]. Based on those previous studies, our QoL cohort followed up 1 year after gastrectomy. Instead, we focused on consecutive multiple time points during that first year after gastrectomy between two groups. In addition to acute change and recovery of QOL after surgery, long term changes of QOL due to gastrointestinal adaption could be further investigated in the future.

In this study, TLTG only determined a better QoL for dysphagia, eating, or odynophagia. DST without purse-string suture was first introduced in 1994 as an easier alternative technique to single stapling technique (SST), but has a risk of high postoperative anastomotic stenosis rate [35, 36]. To overcome this limitation, hDST was proposed, but previous studies still reported high rates of stenosis (7.3–21%) and leakage (4.9–9.9%) [35, 37, 38]. Since 2013, SNUH started TLTG with intracorporeal hDST using needle-guided anvil fixation. Our institution standardized this technique with repeated discussion and consensus among surgeons from the first case, by which all complications were not different among operators (*P* = 0.947, supplementary Table 4). Many previous reports of hDST techniques revealed that it was difficult to tighten the entry hole of the anvil spike at the esophageal stump [37, 38]. On the other hand, the anvil spike located in the middle of the esophageal wall still might have a risk of double stapling across the efferent loop of the Roux limb [39]. As a simple modification, we pulled out the anvil in the medial esophageal wall using the guiding thread, which led to the smallest entry hole for the anvil spike, and secured hDST with a single stapling site completely toward the efferent loop and double stapling site toward the blind loop of the jejunal Roux-limb. The low rates of early and delayed anastomotic complications demonstrated the safety and efficacy of our modified hDST. In the current study, 25 mm circular stapler was routinely used for esophagojejunostomy. Although circular stapler with 28 or 29 mm might be considered to minimize stenosis of esophagojejunostomy, previous nationwide phase II clinical trial for laparoscopic total gastrectomy revealed that ninety-eight patients who underwent laparoscopic total gastrectomy with a 25 mm circular stapler presented no stenosis [21]. On the other hand, it would be often very difficult to insert the shaft of a 28/29 mm circular stapler into jejunal lumen because of its large diameter, and may increase the risk of anastomotic failure.

In addition, the TLTG group with hDST showed better QoL of dysphagia and eating restriction than the LATG group. Adhesion after initial abdominal surgery occurs within the postoperative 1 year and may last for several years [40–43].. Adhesion tissue were reported to contain nerve conducting pain stimuli [44]. Since, fixed adhesion can compromise the lumen of bowel, and filmy adhesion allowing movement between the bowel and surrounding structure can elicit nonobstructive abdominal pain, patients with peritoneal adhesion can manifest vague to highly distressing pain [45]. The unpredictability of abdominal pain caused by adhesions significantly impacts on a patient’s emotions and social life, including fear for eating [46]. We assume that less exposure of the peritoneal cavity, especially the upper abdomen, may induce less adhesion around the anastomosis or provide better recovery of bowel movement [47–49]. Taken together, not only the possible different exposure of peritoneal cavity but also the different anastomotic technique may explain the different QoL in this study.

In this study, we analyzed the CUSUM and learning curve based on the CCI, rather than operation time. In the past, standardizing multiple complications into a single variable seemed impossible due to the absence of adequate methods. However, through introducing CCI, one representative complication index per patient can be estimated. To our knowledge, this is the first study to evaluate the learning curve using CCI. Because CCI is directly related to the patient’s outcome, this approach to the learning curve is more reasonable and intuitively understandable, than previous ones based on operation time. Our study can imply that simple effort to shorten the operation time may be less meaningful during the adoption and stabilization of a novel and complex surgical technique.

This study has some limitations. Firstly, since LATG and TLTG were performed in different time periods, there might be a discrepancy in laparoscopic surgical skills or chronologic changes in clinicopathologic factors between the TLTG and LATG groups. This time trend was inevitable when comparing old and new surgical techniques in retrospective analysis. To minimize this bias, we included all patients in the TLTG group from the first case, and all patients in the LATG group during the same period of TLTG for analysis. Besides, all surgeons at SNUH started performing TLTG in a similar period. Secondly, the sample size for QoL evaluation between TLTG and LATG was limited. In SNUH, an independent prospective cohort study was conducted to analyze only QoL of patients with gastrectomy, regardless of the purpose of the present study. When the current study was designed, the QoL survey for the independent prospective trial were already completed. Totally 84 patients in the current study had pertained QoL data recorded from an independent QoL cohort, and selection process were not based on specific criteria or intentions. Therefore, the independence of QoL data can be the unbiased evidence for current study. Despite the small sample size of QoL data, this is the first study comparing QoL over consecutive multiple time points during the year after operation between TLTG and LATG groups. Considering that a small sample size usually has a risk of yielding false-negative or low sensitivity results, the significant difference in QoL between TLTG and LATG, even in the multivariate analysis, still can be valuable. However, large-scale prospective RCTs are necessary to validate more robust evidence for QoL differences. Thirdly, this study did not include the linear stapling technique for both TLTG and LATG. Previous studies reported a tendency of wider lumen and less stenotic event in linear stapling technique than in circular stapling [50]. One recent study based on PSM analysis revealed that overall postoperative complications including stenotic event in esophagojejunostomy was not different between the two groups [51]. Since our modified hDST also demonstrated the low rate of stenotic complications, prospective randomized clinical trial between the circular stapling including our modified hDST and the linear stapling technique would be expected to provide valuable evidence in the future.

In conclusion, TLTG with hDST were associated with reduced grade I pulmonary complications and better QoL in terms of dysphagia, pain, eating, and odynophagia than LATG for patients with clinical stage I gastric cancer.

## Supplementary Information

**Additional file 1: Supplementary Fig. S1.** Bar graph of annually performed totally laparoscopic total gastrectomy (TLTG) and laparoscopy-assisted total gastrectomy (LATG) cases. **Supplementary Fig. S2.** Patient selection model. **Supplementary Fig. S3.** a. Distribution of propensity score of the cases in the totally laparoscopic total gastrectomy (TLTG) group and laparoscopy-assisted total gastrectomy (LATG) group before and after 2:1 matching. b. Plot of absolute standardized mean differences before and after propensity score matching. c. Dispersion graph of propensity score of the cases in the totally laparoscopic total gastrectomy (TLTG) group and laparoscopy-assisted total gastrectomy (LATG) group before and after 2:1 matching. d. Plot of standardized mean differences before and after propensity score matching. **Supplementary Fig. S4.** The average number of retrieved lymph nodes per each station between totally laparoscopic total gastrectomy (TLTG) group and laparoscopy-assisted total gastrectomy (LATG) group (a) before and (a) after 2:1 matching. **Supplementary Fig. S5.** Cumulative sum (CUSUM) graph using comprehensive complication index (CCI) (a) and operation time (b) over chronological cases in the totally laparoscopic total gastrectomy (TLTG) group. **Supplementary Fig. S6.** Quality of life (QoL) measurement of the totally laparoscopic total gastrectomy (TLTG) group (*n* = 63) and laparoscopy-assisted total gastrectomy (LATG) group (*n* = 21) using the Korean version of European Organization for Research and Treatment of Cancer (EORTC).

**Additional file 2: Supplementary Methods.**

**Additional file 3: Supplementary Table 1.** Operative outcomes and surgical complications of the totally laparoscopic total gastrectomy (TLTG) group compared before and after overcoming the learning curve based on the 26th case. **Supplementary Table 2.** Clinicopathologic characteristics of patients with data of quality of life between the totally laparoscopic total gastrectomy (TLTG) group and laparoscopy-assisted total gastrectomy (LATG) group. **Supplementary Table 3.** Linear regression analysis for variables determining the differences of symptom scale at 6 and 12 months between the totally laparoscopic total gastrectomy (TLTG) group (*n* = 63) and laparoscopy-assisted total gastrectomy (LATG) group (*n* = 21) by backward stepwise methods. **Supplementary Table 4.** Surgical complication of each operator in totally laparoscopic total gastrectomy (TLTG) group.

## Data Availability

The datasets generated and/or analyzed during the current study are not publicly available due to the governmental policy regarding the individual information, but are available from the corresponding author upon reasonable request.

## Abbreviations

QoL: Quality of life
TLTG: Totally laparoscopic total gastrectomy
LATG: Laparoscopy-assisted total gastrectomy
PSM: Propensity score matching
EORTC: European organization for research and treatment of cancer
EGC: Early gastric cancer
KLASS: Korean laparoendoscopic gastrointestinal surgery study
SNUH: Seoul national university hospital
hDST: Hemi-double stapling technique
BMI: Body mass index
CCI: Comprehensive complication index
SST: Single stapling technique

## References

[1] Information Committee of Korean Gastric Cancer, Association. Korean gastric Cancer association nationwide survey on gastric cancer in 2014. J Gastric Cancer. 2016;16(3):131–140, DOI: 10.5230/jgc.2016.16.3.131. 10.5230/jgc.2016.16.3.131 PMC5065942 27752390

[2] Jeong O, Park YK (2011). Clinicopathological features and surgical treatment of gastric cancer in South Korea: the results of 2009 nationwide survey on surgically treated gastric cancer patients. J Gastric Cancer.

[3] Rawla P, Barsouk A (2019). Epidemiology of gastric cancer: global trends, risk factors and prevention. Prz Gastroenterol.

[4] Hyung WJ, Yang HK, Han SU, Lee YJ, Park JM, Kim JJ, Kwon OK, Kong SH, Kim HI, Lee HJ, Kim W, Ryu SW, Jin SH, Oh SJ, Ryu KW, Kim MC, Ahn HS, Park YK, Kim YH, Hwang SH, Kim JW, Cho GS (2019). A feasibility study of laparoscopic total gastrectomy for clinical stage I gastric cancer: a prospective multicenter phase II clinical trial, KLASS 03. Gastric Cancer.

[5] Chen K, Pan Y, Cai JQ, Wu D, Yan JF, Chen DW, Yu HM, Wang XF (2016). Totally laparoscopic versus laparoscopic-assisted total gastrectomy for upper and middle gastric cancer: a single-unit experience of 253 cases with meta-analysis. World J Surg Oncol.

[6] Gong CS, Kim BS, Kim HS (2017). Comparison of totally laparoscopic total gastrectomy using an endoscopic linear stapler with laparoscopic-assisted total gastrectomy using a circular stapler in patients with gastric cancer: a single-center experience. World J Gastroenterol.

[7] Ito H, Inoue H, Odaka N, Satodate H, Onimaru M, Ikeda H, Takayanagi D, Nakahara K, Kudo SE (2014). Evaluation of the safety and efficacy of esophagojejunostomy after totally laparoscopic total gastrectomy using a trans-orally inserted anvil: a single-center comparative study. Surg Endosc.

[8] Jung YJ, Kim DJ, Lee JH, Kim W (2013). Safety of intracorporeal circular stapling esophagojejunostomy using trans-orally inserted anvil (OrVil) following laparoscopic total or proximal gastrectomy - comparison with extracorporeal anastomosis. World J Surg Oncol..

[9] Umemura A, Koeda K, Sasaki A, Fujiwara H, Kimura Y, Iwaya T, Akiyama Y, Wakabayashi G (2015). Totally laparoscopic total gastrectomy for gastric cancer: literature review and comparison of the procedure of esophagojejunostomy. Asian J Surg.

[10] Yamamoto M, Kawano H, Yamaguchi S, Egashira A, Minami K, Morita M, Sakaguchi Y, Toh Y (2017). Technical and survival risks associated with esophagojejunostomy by laparoscopic total gastrectomy for gastric carcinoma. Surg Laparosc Endosc Percutan Tech.

[11] Woo J, Lee JH, Shim KN, Jung HK, Lee HM, Lee HK (2015). Does the difference of invasiveness between totally laparoscopic distal gastrectomy and laparoscopy-assisted distal gastrectomy lead to a difference in early surgical outcomes? A prospective randomized trial. Ann Surg Oncol.

[12] Gertsen EC, Goense L, Brenkman HJF, van Hillegersberg R (2020). Ruurda JP; Dutch upper gastrointestinal Cancer audit (DUCA) group. Identification of the clinically most relevant postoperative complications after gastrectomy: a population-based cohort study. Gastric Cancer.

[13] Ntutumu R, Liu H, Zhen L, Hu YF, Mou TY, Lin T, I BA, Yu J, Li GX (2016). Risk factors for pulmonary complications following laparoscopic gastrectomy: a single-center study. Medicine (Baltimore).

[14] Yasuda K, Shiraishi N, Etoh T, Shiromizu A, Inomata M, Kitano S (2007). Long-term quality of life after laparoscopy-assisted distal gastrectomy for gastric cancer. Surg Endosc.

[15] Kim YW, Baik YH, Yun YH, Nam BH, Kim DH, Choi IJ, Bae JM (2008). Improved quality of life outcomes after laparoscopy-assisted distal gastrectomy for early gastric cancer: results of a prospective randomized clinical trial. Ann Surg.

[16] Kawamura H, Takahashi N, Homma S, Minagawa N, Shibasaki S, Takahashi M, Taketomi A (2014). Assessment of postoperative symptoms after laparoscopy-assisted distal gastrectomy for stage I gastric cancer. Int Surg.

[17] Carvajal SH, Mulvihill SJ (1994). Postgastrectomy syndromes: dumping and diarrhea. Gastroenterol Clin N Am.

[18] Huang ZN, Huang CM, Zheng CH, Li P, Xie JW, Wang JB, Lin JX, Lu J, Chen QY, Cao LL, Lin M, Tu RH, Lin JL (2017). Digestive tract reconstruction using isoperistaltic jejunum-later-cut overlap method after totally laparoscopic total gastrectomy for gastric cancer: short-term outcomes and impact on quality of life. World J Gastroenterol.

[19] Guideline Committee of the Korean Gastric Cancer Association (KGCA), Development Working Group & Review Panel (2019). Korean practice guideline for gastric cancer 2018: an evidence-based, multi-disciplinary approach. J Gastric Cancer..

[20] Japanese Gastric Cancer Association (2017). Japanese gastric cancer treatment guidelines 2014 (ver. 4). Gastric Cancer.

[21] Yang HK, Hyung WJ, Han SU, Lee YJ, Park JM, Cho GS, Kwon OK, Kong SH, Kim HI, Lee HJ, Kim W, Ryu SW, Jin SH, Oh SJ, Ryu KW, Kim MC, Ahn HS, Park YK, Kim YH, Hwang SH, Kim JW, Kim JJ (2020). Comparison of surgical outcomes among different methods of esophagojejunostomy in laparoscopic total gastrectomy for clinical stage I proximal gastric cancer: results of a single-arm multicenter phase II clinical trial in Korea, KLASS 03. Surg Endosc.

[22] Dindo D, Demartines N, Clavien PA (2004). Classification of surgical complications: a new proposal with evaluation in a cohort of 6336 patients and results of a survey. Ann Surg.

[23] Kim SM, Youn HG, An JY, Choi YY, Noh SH, Oh SJ, Sohn TS, Kim S (2018). Comparison of open and laparoscopic gastrectomy in elderly patients. J Gastrointest Surg.

[24] Tu RH, Lin JX, Li P, Xie JW, Wang JB, Lu J, Chen QY, Cao LL, Lin M, Zheng CH, Huang CM (2017). Prognostic significance of postoperative pneumonia after curative resection for patients with gastric cancer. Cancer Med.

[25] Chen K, Xu XW, Zhang RC, Pan Y, Wu D, Mou YP (2013). Systematic review and meta-analysis of laparoscopy-assisted and open total gastrectomy for gastric cancer. World J Gastroenterol.

[26] Liu D, Liang L, Liu L, Zhu Z, Liu S, Hu L, He Y, Fang Y, Wan X (2020). Short-term outcomes and prognosis of laparoscopy-assisted total gastrectomy in elderly patients with stomach cancer. Surg Endosc.

[27] Ford GT, Rosenal TW, Clergue F, Whitelaw WA (1993). Respiratory physiology in upper abdominal surgery. Clin Chest Med.

[28] Desai PM (1999). Pain management and pulmonary dysfunction. Crit Care Clin.

[29] Généreux V, Chassé M, Girard F, Massicotte N, Chartrand-Lefebvre C, Girard M (2020). Effects of positive end-expiratory pressure/recruitment manoeuvres compared with zero end-expiratory pressure on atelectasis during open gynaecological surgery as assessed by ultrasonography: a randomised controlled trial. Br J Anaesth.

[30] Tusman G, Böhm SH, Warner DO, Sprung J (2012). Atelectasis and perioperative pulmonary complications in high-risk patients. Curr Opin Anaesthesiol.

[31] Lee JH, Lee HJ, Choi YS, Kim TH, Huh YJ, Suh YS, Kong SH, Yang HK (2016). Postoperative quality of life after total gastrectomy compared with partial gastrectomy: longitudinal evaluation by European Organization for Research and Treatment of Cancer-OG25 and STO22. J Gastric Cancer..

[32] Avery K, Hughes R, McNair A, Alderson D, Barham P, Blazeby J (2010). Health-related quality of life and survival in the 2 years after surgery for gastric cancer. Eur J Surg Oncol.

[33] Yu W, Park KB, Chung HY, Kwon OK, Lee SS (2016). Chronological changes of quality of life in long-term survivors after Gastrectomy for gastric Cancer. Cancer Res Treat.

[34] Brenkman HJF, Tegels JJW, Ruurda JP, Luyer MDP, Kouwenhoven EA, Draaisma WA, van der Peet DL, Wijnhoven BPL, Stoot J, van Hillegersberg R (2018). Factors influencing health-related quality of life after gastrectomy for cancer. Gastric Cancer.

[35] Zuiki T, Hosoya Y, Kaneda Y, Kurashina K, Saito S, Ui T, Haruta H, Hyodo M, Sata N, Lefor AT, Yasuda Y (2013). Stenosis after use of the double-stapling technique for reconstruction after laparoscopy-assisted total gastrectomy. Surg Endosc.

[36] Tejero Cebrián E, Ratia Giménez T, Fernández Fernández L, Tieso Herreros A, Jorge SE (1994). Double-stapling technique for mechanical circular oesophagojejunal anastomosis after total gastrectomy. Br J Surg.

[37] Amisaki M, Kihara K, Endo K, Suzuki K, Nakamura S, Sawata T, Shimizu T (2016). Comparison of single-stapling and hemi-double-stapling methods for intracorporeal esophagojejunostomy using a circular stapler after totally laparoscopic total gastrectomy. Surg Endosc.

[38] Kosuga T, Hiki N, Nunobe S, Ohashi M, Kubota T, Kamiya S, Sano T, Yamaguchi T (2015). Does the single-stapling technique for circular-stapled esophagojejunostomy reduce anastomotic complications after laparoscopic total gastrectomy?. Ann Surg Oncol.

[39] Omori T, Moon JH, Yamamoto K, Yanagimoto Y, Sugimura K, Miyata H, Yano M, Sakon M (2017). A modified efficient purse-string stapling technique (mEST) that uses a new metal rod for intracorporeal esophagojejunostomy in laparoscopic total gastrectomy. Transl Gastroenterol Hepatol.

[40] Boland GM, Weigel RJ (2006). Formation and prevention of postoperative abdominal adhesions. J Surg Res.

[41] Kim SG, Song KY, Lee HH, Kim EY, Lee JH, Jeon HM, Jeon KH, Jin HM, Kim DJ, Kim W, Yoo HM, Kim JG, Park CH (2019). Efficacy of an antiadhesive agent for the prevention of intra-abdominal adhesions after radical gastrectomy: a prospective randomized, multicenter trial. Medicine (Baltimore).

[42] Parker MC (2004). Epidemiology of adhesions: the burden. Hosp Med.

[43] Sakari T, Christersson M, Karlbom U (2020). Mechanisms of adhesive small bowel obstruction and outcome of surgery; a population-based study. BMC Surg.

[44] Sulaiman H, Gabella G, Davis MSc C, Mutsaers SE, Boulos P, Laurent GJ (2001). Presence and distribution of sensory nerve fibers in human peritoneal adhesions. Ann Surg.

[45] Demco L (2004). Pain mapping of adhesions. J Am Assoc Gynecol Laparosc.

[46] Tabibian N, Swehli E, Boyd A, Umbreen A, Tabibian JH (2017). Abdominal adhesions: a practical review of an often overlooked entity. Ann Med Surg (Lond).

[47] Choi SJ, Gong CS, Kim BS, Kim SO, Kim HS (2019). Clinical outcomes of totally laparoscopic total gastrectomy versus open total gastrectomy for remnant gastric cancer. J Minim Invasive Surg.

[48] Ikeda O, Sakaguchi Y, Aoki Y, Harimoto N, Taomoto J, Masuda T, Ohga T, Adachi E, Toh Y, Okamura T, Baba H (2009). Advantages of totally laparoscopic distal gastrectomy over laparoscopically assisted distal gastrectomy for gastric cancer. Surg Endosc.

[49] Kim BS, Yook JH, Choi YB, Kim KC, Kim MG, Kim TH, Kawada H, Kim BS (2011). Comparison of early outcomes of intracorporeal and extracorporeal gastroduodenostomy after laparoscopic distal gastrectomy for gastric cancer. J Laparoendosc Adv Surg Tech A.

[50] Lee S, Lee H, Song JH, Choi S, Cho M, Son T, Kim HI, Hyung WJ (2020). Intracorporeal esophagojejunostomy using a linear stapler in laparoscopic total gastrectomy: comparison with circular stapling technique. BMC Surg.

[51] Kang SH, Cho YS, Min SH, Park YS, Ahn SH, Park DJ, Kim HH (2019). Intracorporeal Esophagojejunostomy using a circular or a linear stapler in totally laparoscopic Total Gastrectomy: a propensity-matched analysis. J Gastric Cancer.

