# Supplementary material for: Postoperative morbidity and quality of life between totally laparoscopic total gastrectomy and laparoscopy-assisted total gastrectomy: a propensity-score matched analysis
Source: BMC Cancer. 2021 Sep 11;21:1016. doi: 10.1186/s12885-021-08744-1 (PMC8436526; doi:10.1186/s12885-021-08744-1)
Supplement: Supplementary file 1 — Additional file 1: Supplementary Fig. S1. Bar graph of annually performed totally laparoscopic total gastrectomy (TLTG) and laparoscopy-assisted total gastrectomy (LATG) cases. Supplementary Fig. S2. Patient selection model. Supplementary Fig. S3. a. Distribution of propensity score of the cases in the totally laparoscopic total gastrectomy (TLTG) group and laparoscopy-assisted total gastrectomy (LATG) group before and after 2:1 matching. b. Plot of absolute standardized mean differences before and after propensity score matching. c. Dispersion graph of propensity score of the cases in the totally laparoscopic total gastrectomy (TLTG) group and laparoscopy-assisted total gastrectomy (LATG) group before and after 2:1 matching. d. Plot of standardized mean differences before and after propensity score matching. Supplementary Fig. S4. The average number of retrieved lymph nodes per each station between totally laparoscopic total gastrectomy (TLTG) group and laparoscopy-assisted total gastrectomy (LATG) group (a) before and (a) after 2:1 matching. Supplementary Fig. S5. Cumulative sum (CUSUM) graph using comprehensive complication index (CCI) (a) and operation time (b) over chronological cases in the totally laparoscopic total gastrectomy (TLTG) group. Supplementary Fig. S6. Quality of life (QoL) measurement of the totally laparoscopic total gastrectomy (TLTG) group (n = 63) and laparoscopy-assisted total gastrectomy (LATG) group (n = 21) using the Korean version of European Organization for Research and Treatment of Cancer (EORTC). [file 12885_2021_8744_MOESM1_ESM.pdf]

Supplementary Figures

Postoperative morbidity and quality of life between totally laparoscopic total gastrectomy and laparoscopy-assisted total gastrectomy: A propensity-score matched analysis

Journal Name: ***BMC Cancer***

Shin-Hoo Park, M.D.[1,2,3], Yun-Suhk Suh, M.D., Ph.D.[1,2,3], Tae-Han Kim, M.D., Ph.D.[2,5], Yoon-Hee Choi, M.D., Ph.D.[6], Jong-Ho Choi, M.D., Ph.D.[2], Seong-Ho Kong, M.D., Ph.D.[1,2], Do Joong Park, M.D., Ph.D.[1,2], Hyuk-Joon Lee, M.D., Ph.D.[1,2,7], and Han-Kwang Yang, M.D., Ph.D.[1,2,7]

[1]Department of Surgery, Seoul National University College of Medicine, Seoul, Korea

[2]Department of Surgery, Seoul National University Hospital, Seoul, Korea

[3]Department of Foregut Surgery, Korea University Anam Hospital, Seoul, Korea.

[4]Department of Surgery, Seoul National University Bundang Hospital, Seoul, Korea

[5]Department of Surgery, Gyeongsang National University Changwon Hospital, Korea

[6]Division of Medical Statistics, Medical Research Collaborating Center, Seoul National University Hospital, Seoul, Korea

[7]Cancer Research Institute, Seoul National University College of Medicine, Seoul, Korea

Corresponding author: Yun-Suhk Suh

Department of Surgery, Seoul National University College of Medicine

Department of Surgery, Seoul National University Hospital, Seoul, Korea

Department of Surgery, Seoul National University Bundang Hospital, Seoul, Korea

137-82 Gumiro, Bundang-gu, Seongnam-si, Gyeonggi-do 13620, Korea

Tel: +82-31-787-7125

FAX: +82-31-787-4078

E-mail: ysksuh@gmail.com

Supplementary Figure S1. Bar graph of annually performed totally laparoscopic total gastrectomy (TLTG) and laparoscopy-assisted total gastrectomy (LATG) cases.

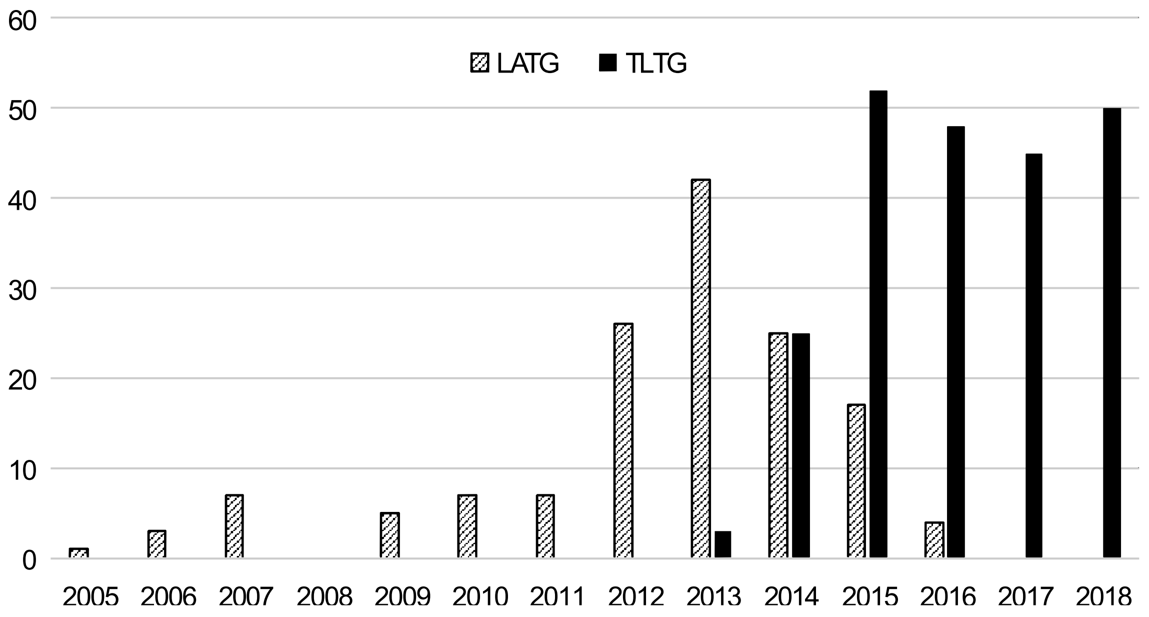

Supplementary Figure S2. Patient selection model.

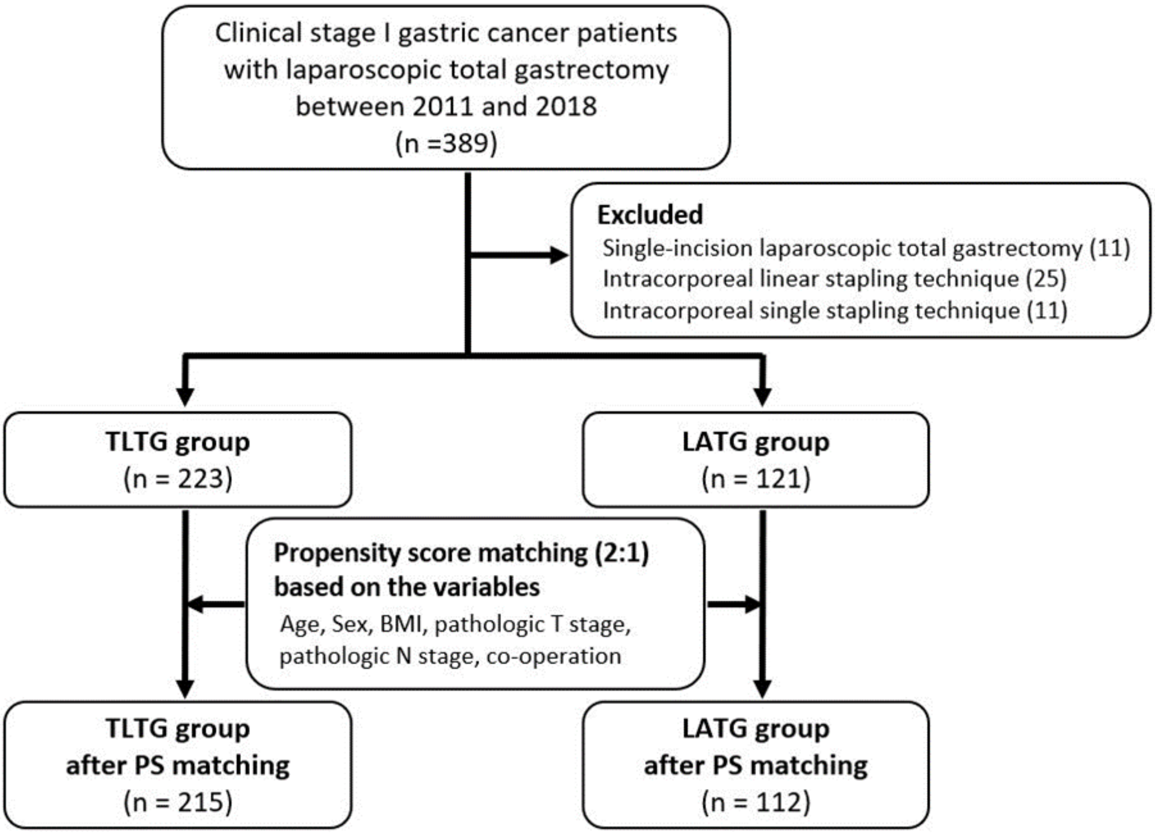

Supplementary Figure S3.

a. Distribution of propensity score of the cases in the totally laparoscopic total gastrectomy (TLTG) group and laparoscopy-assisted total gastrectomy (LATG) group before and after 2:1 matching.

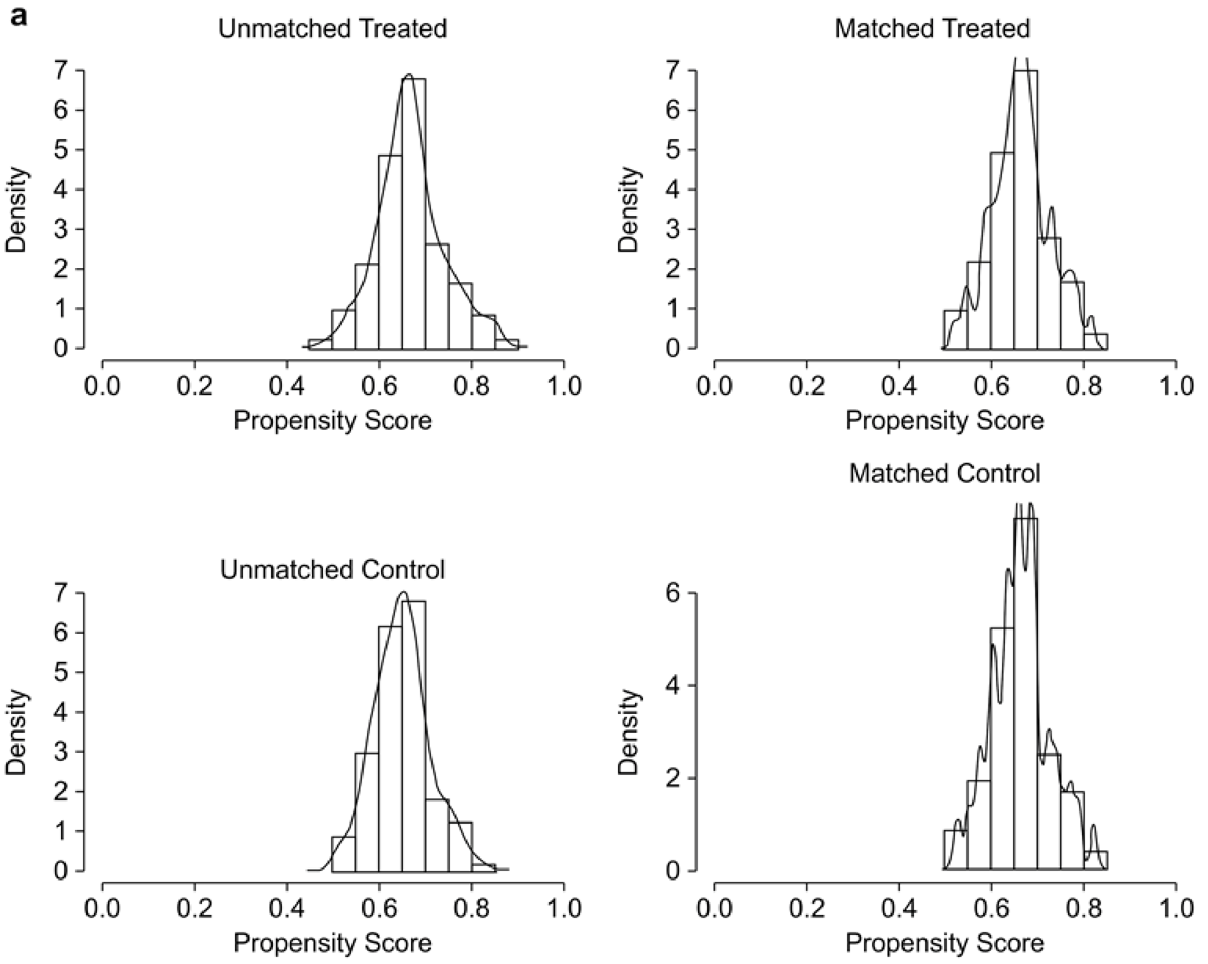

b. Plot of absolute standardized mean differences before and after propensity score matching.

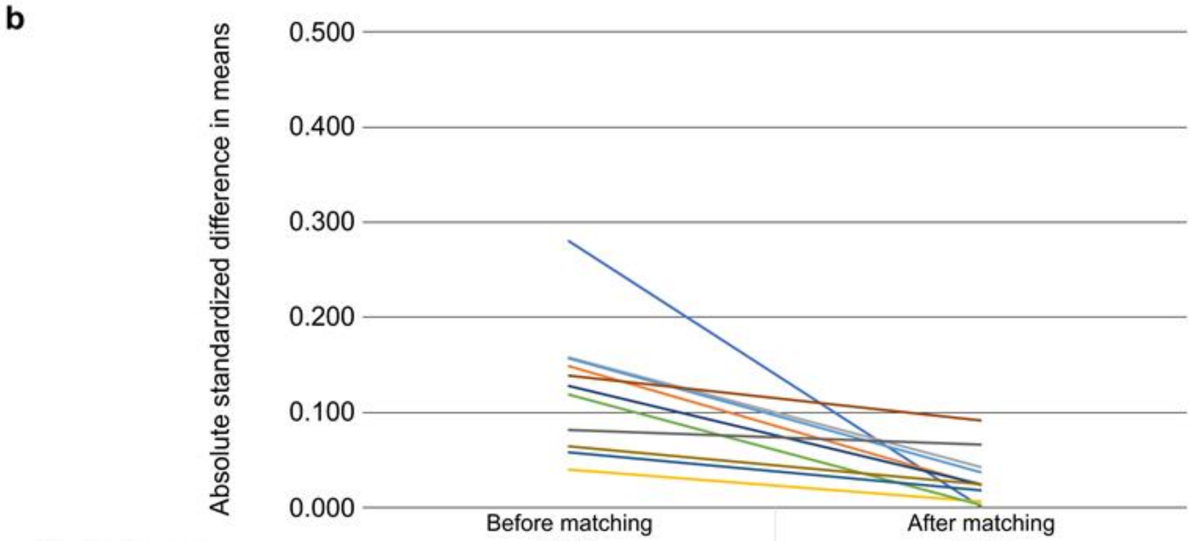

	Before matching	After matching
Propensity score	0.280	0.001
Age	0.149	0.024
Sex	0.158	0.043
Body mass index (kg/m2)	0.040	0.007
Combined organ resection	0.157	0.037
pT stage	0.119	0.003
pN stage	0.128	0.025
TNM stage	0.139	0.092
Tumor size (mm)	0.082	0.066
Number of retrieved lymph nodes	0.065	0.025
Number of metastatic lymph nodes	0.058	0.018

The formula of standardized mean differences (d).

For continuous variable, (d) is defined as d = $\frac{(X_{treated} - X_{control})}{\sqrt{\frac{S^2{}_{treated} + S^2{}_{control}}{2}}}$, where $X_{treated}$ and $X_{control}$ are sample means of the variable in intervention and control groups, respectively, and $S^2{}_{treated}$ and $S^2{}_{control}$ are sample variances of the variable in the respective groups.

For the binary variable, (d) is defined as d = $\frac{(P_{treated} - P_{control})}{\sqrt{\frac{P_{treated}(1-P_{treated}) + P_{control}(1-P_{control})}{2}}}$, where $P_{treated}$ and $P_{control}$ are sample proportions for variable in the treated and control groups, respectively.

c. Dispersion graph of propensity score of the cases in the totally laparoscopic total gastrectomy (TLTG) group and laparoscopy-assisted total gastrectomy (LATG) group before and after 2:1 matching.

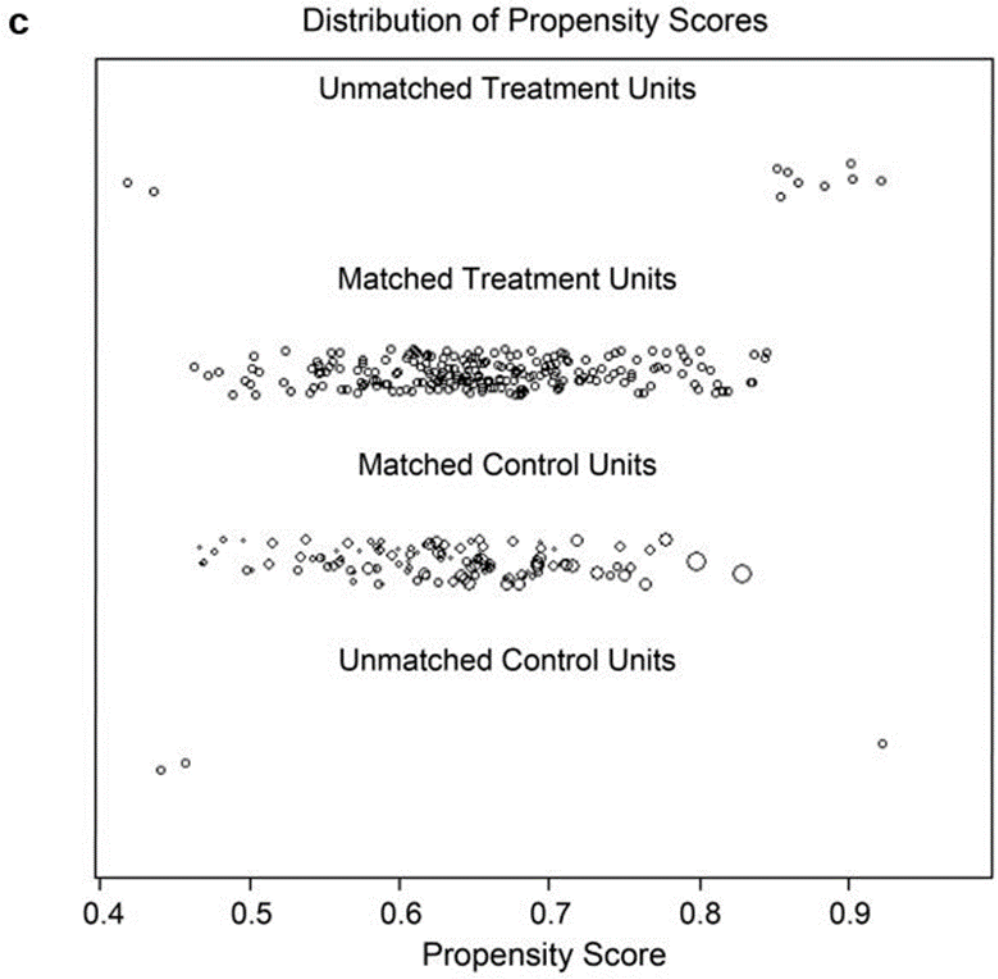

d. Plot of standardized mean differences before and after propensity score matching.

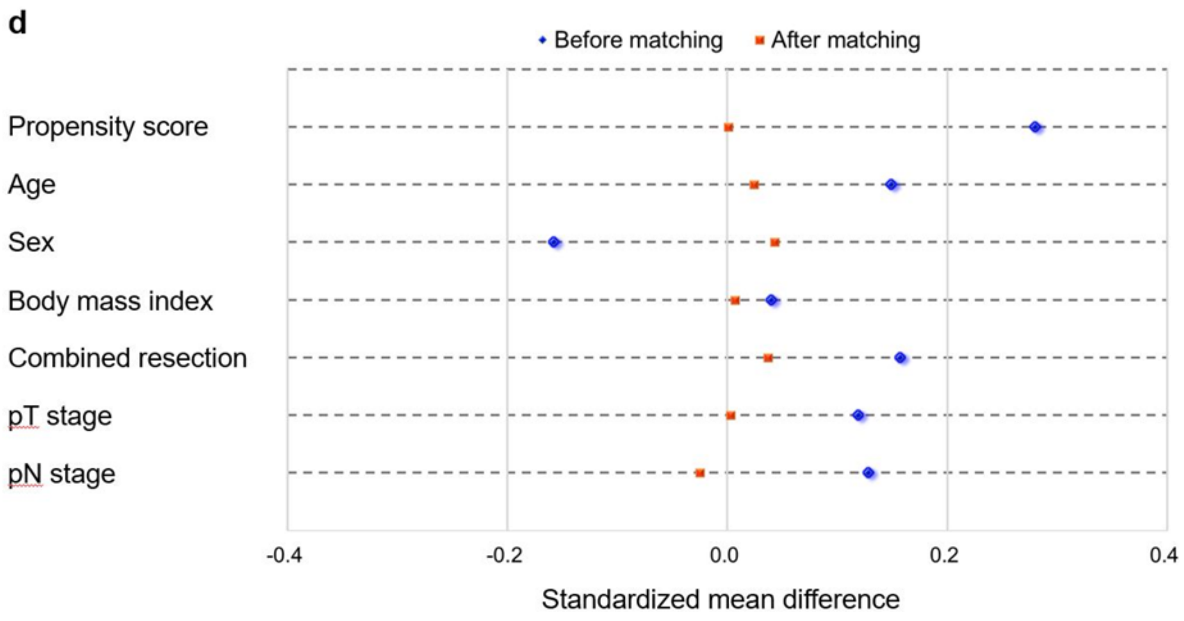

Supplementary Figure S4. The average number of retrieved lymph nodes per each station between totally laparoscopic total gastrectomy (TLTG) group and laparoscopy-assisted total gastrectomy (LATG) group (a) before and (a) after 2:1 matching.

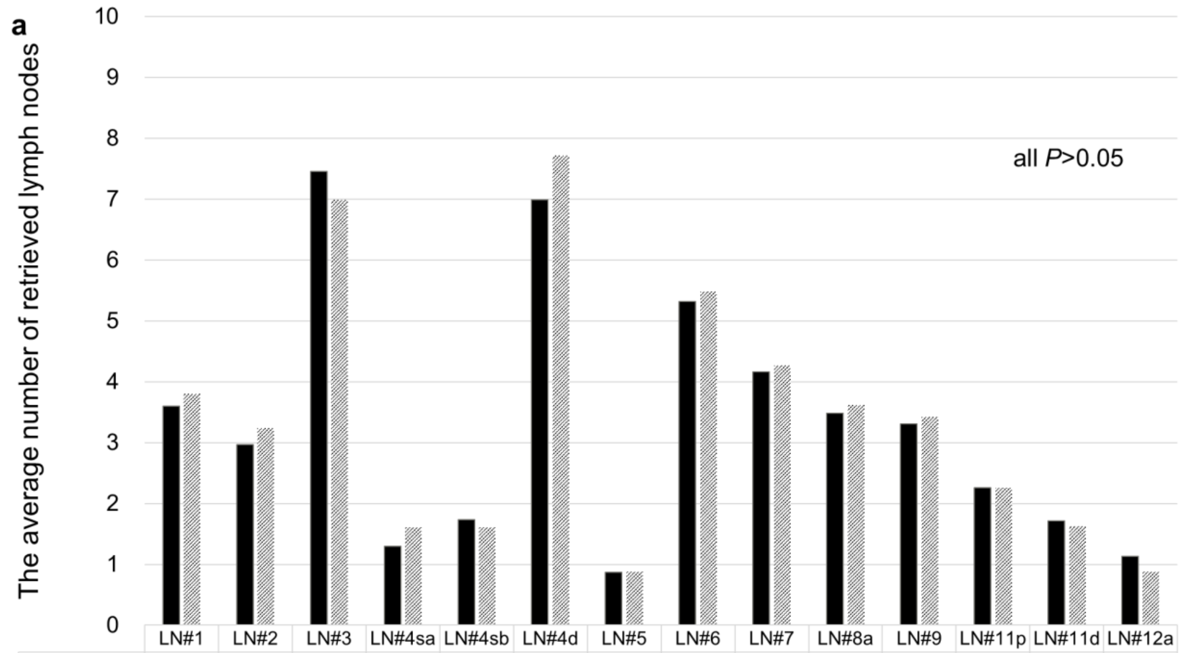

	LN#1	LN#2	LN#3	LN#4sa	LN#4sb	LN#4d	LN#5	LN#6	LN#7	LN#8a	LN#9	LN#11p	LN#11d	LN#12a
■ TLTG group	3.6	3.0	7.5	1.3	1.7	7.0	0.9	5.3	4.2	3.5	3.3	2.3	1.7	1.1
▨ LATG group	3.8	3.2	7.0	1.6	1.6	7.7	0.9	5.5	4.3	3.6	3.4	2.3	1.6	0.9

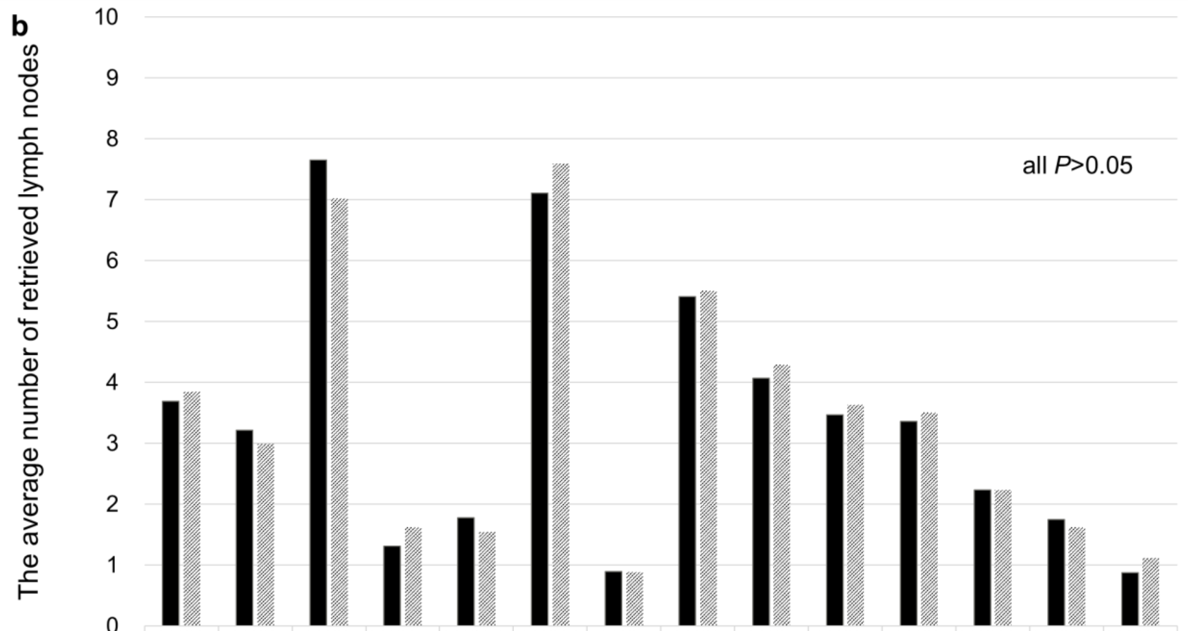

	LN#1	LN#2	LN#3	LN#4sa	LN#4sb	LN#4d	LN#5	LN#6	LN#7	LN#8a	LN#9	LN#11p	LN#11d	LN#12a
■ TLTG group	3.7	3.2	7.7	1.3	1.8	7.1	0.9	5.4	4.1	3.5	3.4	2.2	1.8	0.9
▨ LATG group	3.9	3.0	7.0	1.6	1.6	7.6	0.9	5.5	4.3	3.6	3.5	2.2	1.6	1.1

Supplementary Figure S5. Cumulative sum (CUSUM) graph using comprehensive complication index (CCI) (a) and operation time (b) over chronological cases in the totally laparoscopic total gastrectomy (TLTG) group.

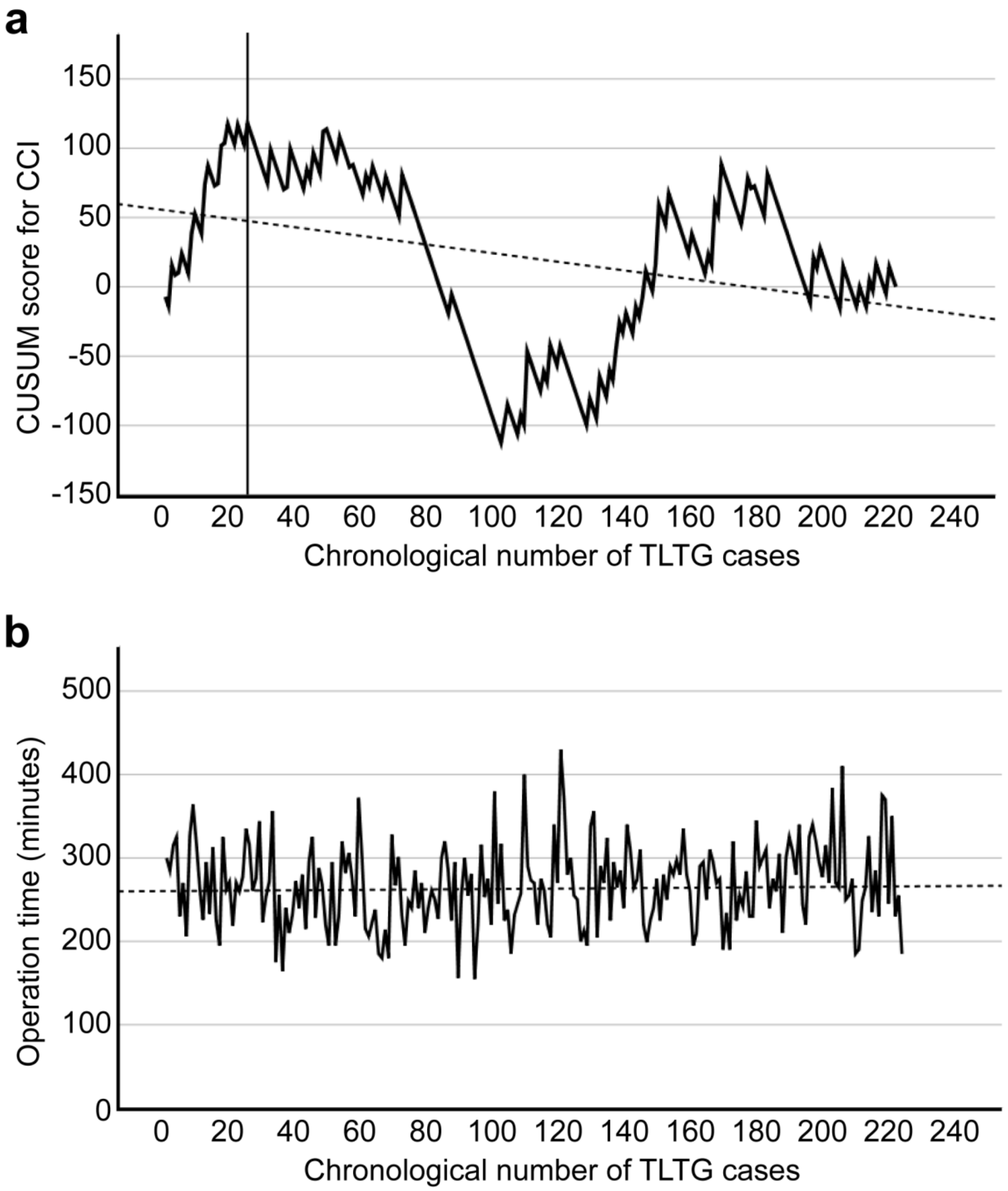

Supplementary Figure S6. Quality of life (QoL) measurement of the totally laparoscopic total gastrectomy (TLTG) group (n=63) and laparoscopy-assisted total gastrectomy (LATG) group (n=21) using the Korean version of European Organization for Research and Treatment of Cancer (EORTC).

a. Global status

b. Functional scale

c. Symptom scale

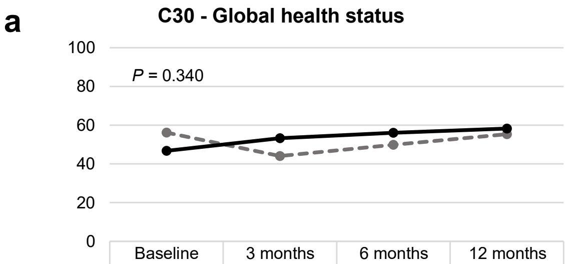

	Baseline	3 months	6 months	12 months
LATG	56.19	44.07	49.83	55.33
TLTG	46.74	53.30	56.12	58.25

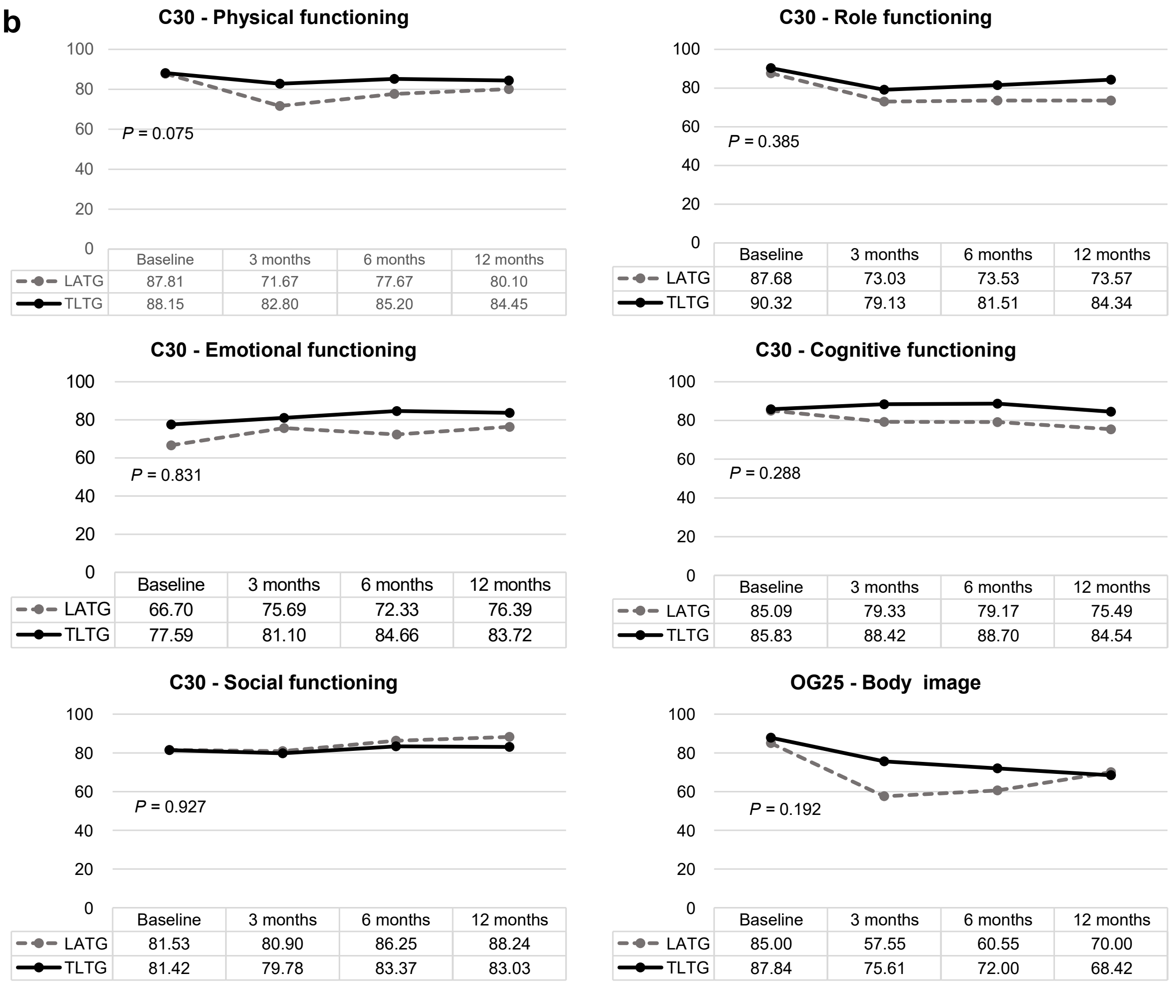
b
C30 - Physical functioning
P = 0.075
Baseline 3 months 6 months 12 months
LATG 87.81 71.67 77.67 80.10
TLTG 88.15 82.80 85.20 84.45
C30 - Role functioning
P = 0.385
Baseline 3 months 6 months 12 months
LATG 87.68 73.03 73.53 73.57
TLTG 90.32 79.13 81.51 84.34
C30 - Emotional functioning
P = 0.831
Baseline 3 months 6 months 12 months
LATG 66.70 75.69 72.33 76.39
TLTG 77.59 81.10 84.66 83.72
C30 - Cognitive functioning
P = 0.288
Baseline 3 months 6 months 12 months
LATG 85.09 79.33 79.17 75.49
TLTG 85.83 88.42 88.70 84.54
C30 - Social functioning
P = 0.927
Baseline 3 months 6 months 12 months
LATG 81.53 80.90 86.25 88.24
TLTG 81.42 79.78 83.37 83.03
OG25 - Body image
P = 0.192
Baseline 3 months 6 months 12 months
LATG 85.00 57.55 60.55 70.00
TLTG 87.84 75.61 72.00 68.42

c.1

C30 - Fatigue

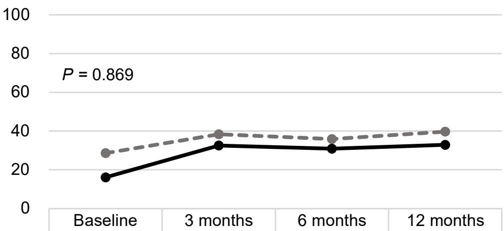

	Baseline	3 months	6 months	12 months
LATG	28.61	38.35	35.86	39.72
TLTG	16.14	32.53	30.89	32.91

C30 - Nausea and vomiting

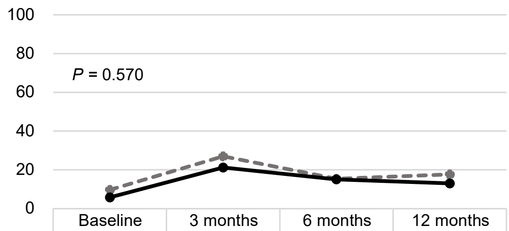

	Baseline	3 months	6 months	12 months
LATG	9.65	27.00	15.36	17.71
TLTG	5.80	21.22	15.05	12.97

C30 - Pain

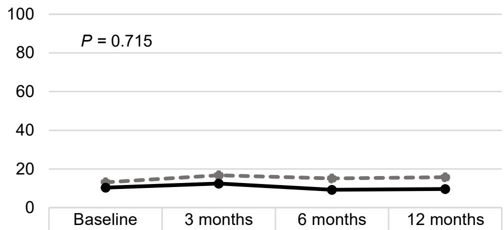

	Baseline	3 months	6 months	12 months
LATG	13.09	16.72	15.14	15.71
TLTG	10.32	12.46	9.24	9.59

C30 - Dyspnea

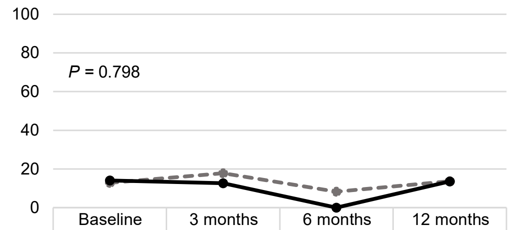

	Baseline	3 months	6 months	12 months
LATG	12.85	17.79	8.25	13.66
TLTG	14.04	12.62	0	13.54

C30 - Insomnia

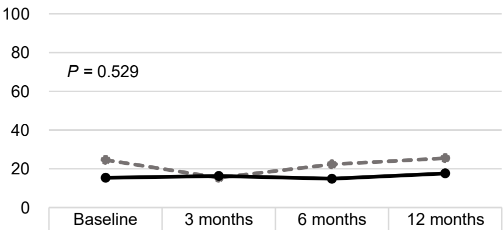

	Baseline	3 months	6 months	12 months
LATG	24.53	15.38	22.25	25.45
TLTG	15.31	16.31	14.88	17.59

C30 - Appetite loss

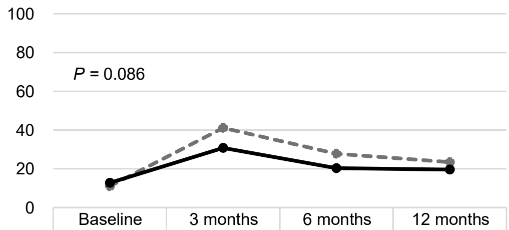

	Baseline	3 months	6 months	12 months
LATG	11.00	41.10	27.75	23.47
TLTG	12.74	30.78	20.31	19.63

C30 - Constipation

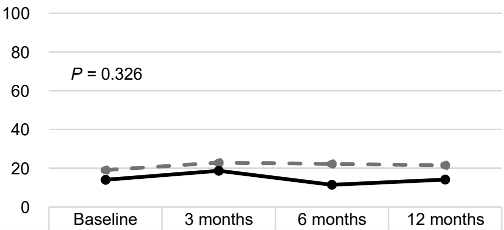

	Baseline	3 months	6 months	12 months
LATG	19.06	22.89	22.17	21.47
TLTG	14.03	18.69	11.45	14.19

C30 - Diarrhea

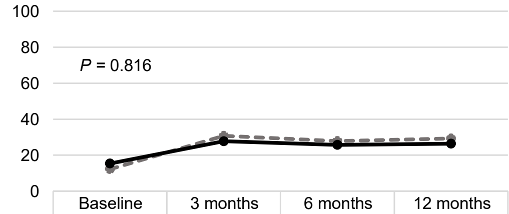

	Baseline	3 months	6 months	12 months
LATG	12.19	30.75	27.78	29.29
TLTG	15.34	27.76	25.71	26.42

c.2

C30 - Financial difficulties

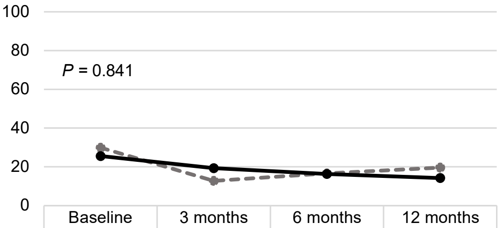

	Baseline	3 months	6 months	12 months
LATG	29.88	12.77	16.67	19.61
TLTG	25.57	19.31	16.27	14.2

OG25 - Dysphagia

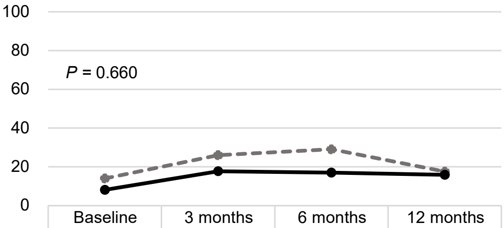

	Baseline	3 months	6 months	12 months
LATG	14.00	26.00	29.09	17.60
TLTG	8.11	17.73	17.06	15.91

OG25 - Reflux

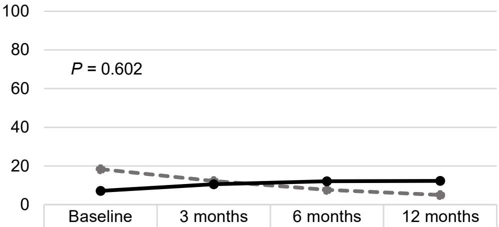

	Baseline	3 months	6 months	12 months
LATG	18.36	12.18	7.63	5.00
TLTG	7.11	10.55	12.12	12.27

OG25 - Pain and Discomfort

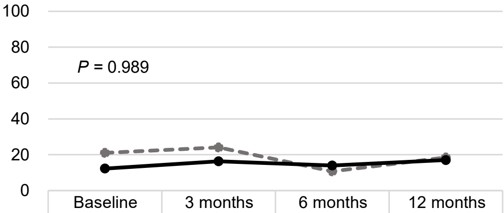

	Baseline	3 months	6 months	12 months
LATG	21.00	24.09	10.73	18.50
TLTG	12.26	16.29	13.94	17.04

OG25 - Anxiety

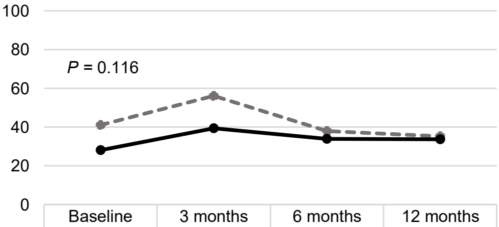

	Baseline	3 months	6 months	12 months
LATG	41.00	56.09	37.91	35.20
TLTG	28.05	39.35	33.90	33.64

OG25 - Eating with others

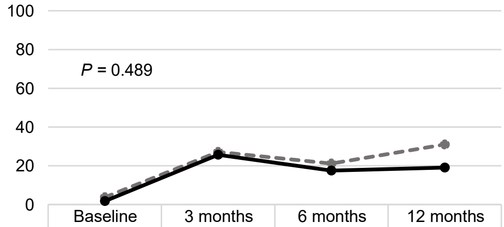

	Baseline	3 months	6 months	12 months
LATG	3.72	27.18	21.18	31.01
TLTG	1.74	25.67	17.51	19.09

OG25 - Dry mouth

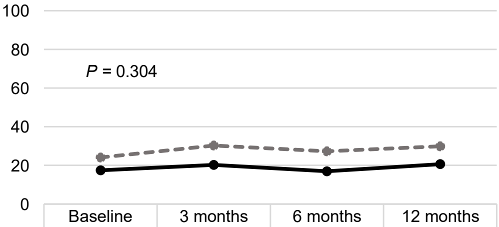

	Baseline	3 months	6 months	12 months
LATG	24.09	30.27	27.27	29.90
TLTG	17.42	20.29	16.96	20.62

OG25 - Trouble with Taste

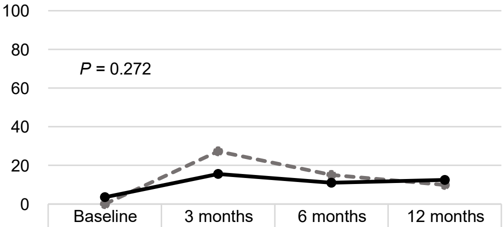

	Baseline	3 months	6 months	12 months
LATG	0.00	27.27	15.09	9.90
TLTG	3.53	15.55	11.06	12.49

c.3

OG25 - Trouble with swallowing saliva

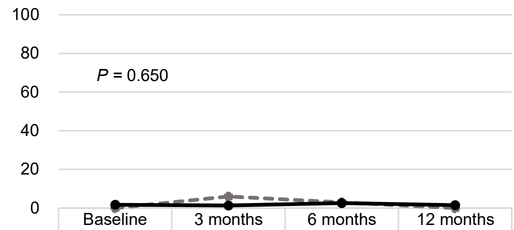

	Baseline	3 months	6 months	12 months
LATG	0.00	6.00	3.00	0.00
TLTG	1.74	1.35	2.59	1.47

OG25 - Trouble with Talking

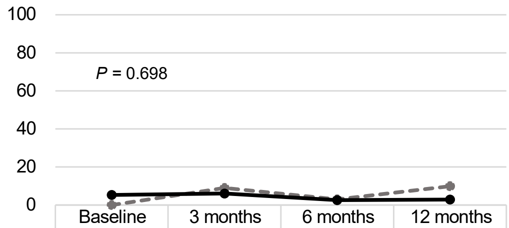

	Baseline	3 months	6 months	12 months
LATG	0.00	9.00	3.00	9.90
TLTG	5.26	6.08	2.59	2.93

OG25 - Trouble with Coughing

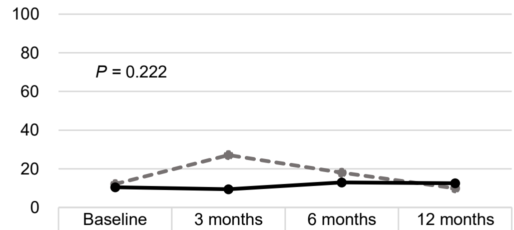

	Baseline	3 months	6 months	12 months
LATG	12.09	27.09	18.00	9.90
TLTG	10.42	9.43	12.94	12.47

OG25 - Choked when swallowing

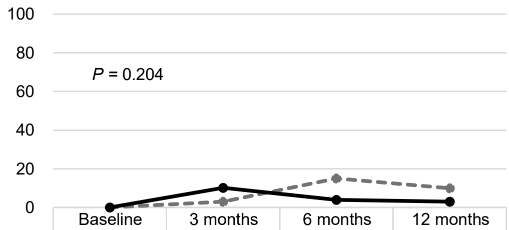

	Baseline	3 months	6 months	12 months
LATG	0.00	3.00	15.00	9.90
TLTG	0.00	10.16	3.88	2.96

OG25 - Weight loss

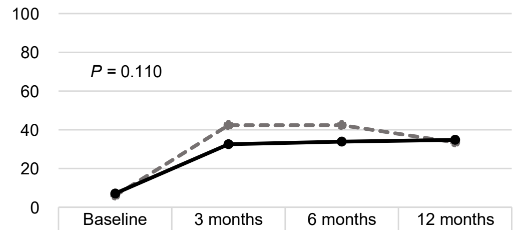

	Baseline	3 months	6 months	12 months
LATG	6.00	42.36	42.36	33.50
TLTG	7.11	32.57	33.88	34.78

OG25 - Hair loss

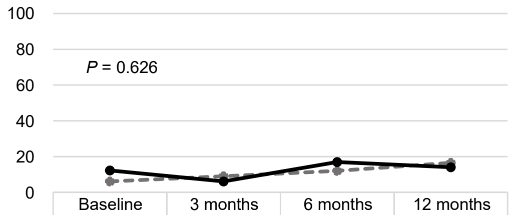

	Baseline	3 months	6 months	12 months
LATG	6.09	9.00	12.00	16.50
TLTG	12.21	6.10	16.94	14.00
