# Supplementary material for: Postoperative morbidity and quality of life between totally laparoscopic total gastrectomy and laparoscopy-assisted total gastrectomy: a propensity-score matched analysis
Source: BMC Cancer. 2021 Sep 11;21:1016. doi: 10.1186/s12885-021-08744-1 (PMC8436526; doi:10.1186/s12885-021-08744-1)
Supplement: Supplementary file 2 — Additional file 2: Supplementary Methods. [file 12885_2021_8744_MOESM2_ESM.pdf]

Supplementary Methods

Postoperative morbidity and quality of life between totally laparoscopic total gastrectomy and laparoscopy-assisted total gastrectomy: A propensity-score matched analysis

Journal Name: ***BMC Cancer***

Shin-Hoo Park, M.D.[1,2,3], Yun-Suhk Suh, M.D., Ph.D.[1,2,4], Tae-Han Kim, M.D., Ph.D.[2,5], Yoon-Hee Choi, M.D., Ph.D.[6], Jong-Ho Choi, M.D., Ph.D.[2], Seong-Ho Kong, M.D., Ph.D.[1,2], Do Joong Park, M.D., Ph.D.[1,2], Hyuk-Joon Lee, M.D., Ph.D.[1,2,7], and Han-Kwang Yang, M.D., Ph.D.[1,2,7]

[1]Department of Surgery, Seoul National University College of Medicine, Seoul, Korea.

[2]Department of Surgery, Seoul National University Hospital, Seoul, Korea.

[3]Department of Foregut Surgery, Korea University Anam Hospital, Seoul, Korea.

[4]Department of Surgery, Seoul National University Bundang Hospital, Seoul, Korea.

[4]Department of Surgery, Gyeongsang National University Changwon Hospital, Korea.

[5]Division of Medical Statistics, Medical Research Collaborating Center, Seoul National University Hospital, Seoul, Korea.

[6]Cancer Research Institute, Seoul National University College of Medicine, Seoul, Korea.

Corresponding author: Yun-Suhk Suh

Department of Surgery, Seoul National University College of Medicine, Seoul, Korea.

Department of Surgery, Seoul National University Hospital, Seoul, Korea.

Department of Surgery, Seoul National University Bundang Hospital, Seoul, Korea.
137-82 Gumiro, Bundang-gu, Seongnam-si, Gyeonggi-do 13620, Korea.
Tel: +82-31-787-7125
FAX: +82-31-787-4078
E-mail: ysksuh@gmail.com

Surgical outcome

To determine the trends of complication data and learning curve, the cumulative sum (CUSUM) score using the CCI and operation time of the TLTG group was calculated. The formula of CUSUM is $\sum_{i=1}^{n}(Xi - \mu)$, where Xi is the CCI or operation time of each case, and μ is the average value of the CCI or operation time in the TLTG group. The time period to overcome the learning curve was determined as follows: Of the cumulative cases with the peak value of CUSUM, if the CCI, overall complication, or operation time shows the greatest reduction after the case compared before, then that case was considered as the time of overcoming the learning curve. The TLTG group after overcoming the learning curve was 2:1 propensity score matched again to control cases of the LATG group [1].

Quality of Life

We reviewed the independently collected QoL cohort of gastric cancer patients in SNUH, which had been recruited irrespective of the original purpose of the current study. The QoL cohort was approved by the Institutional Review Board of SNUH (H1406-108-590). Patients who were scheduled to undergo laparoscopic distal or total gastrectomy and have a willingness to participate the EORTC QoL questionnaires (C30, STO22, and OG25) were included. The QoL survey was followed preoperatively and at 3 months, 6 months, and 1 year postoperatively.

Of these cohorts, we separately retrieved QoL data of patients matched to our TLTG and LATG groups. The difference in QoL trends over time periods between the TLTG and LATG groups was analyzed by repeated measure analysis of variance (ANOVA), by controlling preoperative QoL score as the covariate. Analysis of covariance (ANCOVA) was used to

compare the QoL at certain postoperative time points (3, 6, 12 months) between two groups, by controlling the confounding effect of preoperative QoL score. When the QoL at certain time points (3, 6 or 12 months) were different between two groups, multivariate backward stepwise linear regression analysis was used to determine the most independent risk factors for the differences of QoL at each month, after excluding all possible confounding factors.

Statistical analysis

Fisher's exact test was used for categorical variables. Student's t-test and one-way ANOVA were used to compare continuous variables between the two groups or among more than three groups. Wilcoxon rank sum test was used to compare two groups with the CCI as non-parametric data. All tests were two-sided, and P value of <0.05 was considered statistically significant. Statistical analyses were performed using the IBM SPSS Statistics version 25 (IBM Corp., Armonk, NY).

References

1. Kim TH, Suh YS, Huh YJ, et al. The comprehensive complication index (CCI) is a more sensitive complication index than the conventional Clavien-Dindo classification in radical gastric cancer surgery. Gastric Cancer. 2018;21(1):171-181.
