# Supplementary material for: Postoperative morbidity and quality of life between totally laparoscopic total gastrectomy and laparoscopy-assisted total gastrectomy: a propensity-score matched analysis
Source: BMC Cancer. 2021 Sep 11;21:1016. doi: 10.1186/s12885-021-08744-1 (PMC8436526; doi:10.1186/s12885-021-08744-1)
Supplement: Supplementary file 3 — Additional file 3: Supplementary Table 1. Operative outcomes and surgical complications of the totally laparoscopic total gastrectomy (TLTG) group compared before and after overcoming the learning curve based on the 26th case. Supplementary Table 2. Clinicopathologic characteristics of patients with data of quality of life between the totally laparoscopic total gastrectomy (TLTG) group and laparoscopy-assisted total gastrectomy (LATG) group. Supplementary Table 3. Linear regression analysis for variables determining the differences of symptom scale at 6 and 12 months between the totally laparoscopic total gastrectomy (TLTG) group (n = 63) and laparoscopy-assisted total gastrectomy (LATG) group (n = 21) by backward stepwise methods. Supplementary Table 4. Surgical complication of each operator in totally laparoscopic total gastrectomy (TLTG) group. [file 12885_2021_8744_MOESM3_ESM.pdf]

Supplementary Tables

Postoperative morbidity and quality of life between totally laparoscopic total gastrectomy and laparoscopy-assisted total gastrectomy: A propensity-score matched analysis

Journal Name: ***BMC Cancer***

Shin-Hoo Park, M.D.[1,2,3], Yun-Suhk Suh, M.D., Ph.D.[1,2,4], Tae-Han Kim, M.D., Ph.D.[2,5], Yoon-Hee Choi, M.D., Ph.D.[6], Jong-Ho Choi, M.D., Ph.D.[2], Seong-Ho Kong, M.D., Ph.D.[1,2], Do Joong Park, M.D., Ph.D.[1,2], Hyuk-Joon Lee, M.D., Ph.D.[1,2,7], and Han-Kwang Yang, M.D., Ph.D.[1,2,7]

[1]Department of Surgery, Seoul National University College of Medicine, Seoul, Korea.

[2]Department of Surgery, Seoul National University Hospital, Seoul, Korea.

[3]Department of Foregut Surgery, Korea University Anam Hospital, Seoul, Korea.

[4]Department of Surgery, Seoul National University Bundang Hospital, Seoul, Korea.

[5]Department of Surgery, Gyeongsang National University Changwon Hospital, Korea.

[6]Division of Medical Statistics, Medical Research Collaborating Center, Seoul National University Hospital, Seoul, Korea.

[7]Cancer Research Institute, Seoul National University College of Medicine, Seoul, Korea.

Corresponding author: Yun-Suhk Suh

Department of Surgery, Seoul National University College of Medicine, Seoul, Korea.

Department of Surgery, Seoul National University Hospital, Seoul, Korea.

Department of Surgery, Seoul National University Bundang Hospital, Seoul, Korea.

137-82 Gumiro, Bundang-gu, Seongnam-si, Gyeonggi-do 13620, Korea.

Tel: +82-31-787-7125

FAX: +82-31-787-4078

E-mail: ysksuh@gmail.com

Supplementary Table 1. Operative outcomes and surgical complications of the totally laparoscopic total gastrectomy (TLTG) group compared before and after overcoming the learning curve based on the 26th case.

Variables	Early TLTG (n = 26)	Late TLTG (n = 197)	*P* value
Mean operation time: min (SD)	278.0 ± 44.8	265.9 ± 51.0	0.211
Transfusion	0 (0)	2 (1.6)	0.371
Overall complication: n (%)	13 (50.0)	48 (24.8)	0.009
Major complication (≥grade IIIa)	3 (11.5)	23 (11.7)	0.984
Comprehensive complication index	11.7 (0-42.4)	6.5 (0-60.2)	0.014
Complication detected within 1 month			
Grade I	6 (23.1)	6 (3.0)	0.001
Fluid collection	3 (11.5)	0 (0)	0.001
Motility disorder	1 (3.8)	2 (1.0)	0.312
Ischemia	0 (0)	1 (0.5)	0.716
Other infection	1 (3.8)	0 (0)	0.117
Pulmonary	0 (0)	2 (1.0)	0.606
Other systemic complication	1 (3.8)	1 (0.5)	0.220
Grade II	8 (30.8)	25 (12.7)	0.034
Fluid collection	3 (11.5)	6 (3.0)	0.074
Intra-abdominal bleeding	0 (0)	1 (0.5)	0.716
Luminal bleeding	1 (3.8)	0 (0)	0.117
Ileus/motility disorder	1 (3.8)	1 (0.5)	0.220

Anastomosis site leakage	1 (3.8)	3 (1.5)	0.393
Ischemia	0 (0)	1 (0.5)	0.716
Pulmonary	3 (11.5)	10 (5.1)	0.182
Other systemic complication	1 (3.8)	6 (3.0)	0.585
Grade IIIa	5 (10.2)	19 (10.9)	0.886
Wound	0 (0)	2 (1.0)	0.606
Fluid collection	1 (3.8)	11 (5.6)	0.712
Intra-abdominal bleeding	0 (0)	1 (0.5)	0.716
Stenosis	1 (3.8)	0 (0)	0.117
Motility disorder	0 (0)	1 (0.5)	0.716
Anastomosis site leakage	0 (0)	5 (2.5)	0.411
Other fistula	0 (0)	0 (0)	-
Pulmonary	1 (3.8)	5 (2.5)	0.529
Other systemic complication	0 (0)	1 (0.5)	0.716
Grade IIIb	0 (0)	2 (1.0)	0.606
Wound	0 (0)	2 (1.0)	0.606
Motility disorder	0 (0)	0 (0)	-
Grade IVa	1 (3.8)	2 (1.0)	0.312
Intra-abdominal bleeding	0 (0)	1 (0.5)	0.716
Pulmonary	1 (3.8)	1 (0.5)	0.220
Grade IVb	0 (0)	1 (0.5)	0.716
Intra-abdominal bleeding	0 (0)	1 (0.5)	0.716

Complication detected from 1 month to 1 year

Delayed EJ stenosis (total no.)	2 (7.7)	12 (6.1)	0.670
Delayed EJ stenosis (≥ CD IIIa)	1 (3.8)	6 (3.0)	0.585
Detected period for (days)	103.5 ± 20.5	72.3 ± 19.6	0.244

Abbreviation: EJ = esophagojejunostomy

The rationales for defining the 26th case as a point of overcoming the learning curve.

i) When the TLTG group was divided by the 26th case rather than another peak point, the CCI (6.54±12.65 vs 11.67±14.23, *P*=0.014), overall complication (24.7% vs 50.0%, *P*=0.009), grade I complication (3.0% vs 23.1%, *P*=0.001), and grade I fluid collection (0% vs 11.5%, *P*=0.001) showed a greater reduction in the late TLTG (n= 197) than in the early TLTG (n=26) group.

ii) The late TLTG group had significantly less grade II complications than the early TLTG group, only when the TLTG group was divided by the 26th case rather than other criteria.

iii). When divided by the 26th case, the operation time tended to be shorter in the late TLTG group than in the early TLTG group.

Supplementary Table 2. Clinicopathologic characteristics of patients with data of quality of life between the totally laparoscopic total gastrectomy (TLTG) group and laparoscopy-assisted total gastrectomy (LATG) group.

Variables	TLTG (n = 63)	LATG (n = 21)	*P* value
Age	58.8 ± 9.4	61.9 ± 7.9	0.184
Sex			1.000
Male	45 (71.4)	15 (71.4)	
Female	18 (28.6)	6 (28.6)	
Body mass index (kg/m^2)	24.1 ± 2.5	23.9 ± 2.5	0.728
Underlying disease			
Cerebrovascular disease	4 (6.3)	1 (4.8)	0.790
Dementia	0 (0)	0 (0)	-
Congestive (Ischemic) heart disease	3 (4.8)	2 (9.5)	0.595
Peripheral vascular disease	0 (0)	0 (0)	-
Hypertension	19 (30.2)	7 (33.3)	0.791
Pulmonary disease	5 (7.9)	1 (4.8)	0.625
Diabetes	7 (11.1)	2 (9.5)	0.839
Liver disease	2 (3.2)	0 (0)	0.409
Renal disease	0 (0)	0 (0)	-
Hemi- or paraplesia	0 (0)	0 (0)	-
Rheumatologic disorder	1 (1.6)	0 (0)	0.561
Any malignancies	0 (0)	0 (0)	-

Charlson comorbidity index			
Median [range]	0 [0-3]	0 [0-3]	0.942
0	35 (55.6)	12 (57.1)	0.924
1-2	26 (41.3)	8 (38.1)	
≥3	2 (3.2)	1 (4.8)	
Combined resection			0.409
None	61 (96.8)	21 (100)	
Gallbladder	2 (3.2)	0 (0)	
Spleen	0 (0)	0 (0)	
pT category			0.443
pT1	53 (84.1)	17 (81.0)	
pT2	7 (11.1)	1 (4.8)	
pT3	2 (3.2)	2 (9.5)	
pT4	1 (1.6)	1 (4.8)	
pN category			0.211
pN0	58 (92.1)	18 (85.7)	
pN1	1 (1.6)	1 (4.8)	
pN2	3 (4.8)	0 (0)	
pN3	1 (1.6)	2 (9.5)	
TNM stage*			0.496
Stage I	58 (92.1)	18 (85.7)	
Stage II	3 (4.8)	1 (4.8)	
Stage III	2 (3.2)	2 (9.5)	

Adjuvant chemotherapy	4 (6.3)	1 (4.8)	0.790
Hospital stay (days)	11.2 ± 7.6	11.0 ± 4.5	0.907
Comprehensive complication index	8.0 (0-60.2)	5.3 (0-33.5)	0.587
Overall complication: n (%)	23 (36.5)	6 (28.6)	0.602
Major complication (≥grade IIIa)	7 (11.1)	2 (9.5)	0.839
Anastomotic complication	2 (3.2)	0 (0)	0.409
Motility disorder	2 (3.2)	1 (4.8)	0.734

*TNM stage according to AJCC, the 7th edition.

Supplementary table 3. Linear regression analysis for variables determining the differences of symptom scale at 6 and 12 months between the totally laparoscopic total gastrectomy (TLTG) group (n=63) and laparoscopy-assisted total gastrectomy (LATG) group (n=21) by backward stepwise methods. Matching variables were age, sex, body mass index, TLTG (vs. LATG), pT stage and pN stage, baseline quality of life score, anastomotic complication, motility disorder.

Symptom scale	Variable factors	Unstandardized coefficient		*P* value
		B	Standard errors	
STO22 dysphagia (6 months)	TLTG (vs LATG)	-20.928	5.234	<0.001
STO22 pain (3 months)	TLTG (vs LATG)	-11.635	5.108	0.031
	STO22 pain (preoperative)	0.419	0.202	0.047
STO22 pain (6 months)	TLTG (vs LATG)	-16.975	5.061	0.002
STO22 pain (12 months)	TLTG (vs LATG)	-16.170	4.762	0.002
STO22 eating restriction (6 months)	TLTG (vs LATG)	-11.407	4.983	0.031
	STO22 eating (preoperative)	0.657	0.295	0.035
OG25 eating (6 months)	TLTG (vs LATG)	-11.793	3.969	0.007
	OG25 eating (preoperative)	0.663	0.230	0.008
OG25 eating (12 months)	TLTG (vs LATG)	-16.641	6.010	0.010
OG25 odynophagia (6 months)	TLTG (vs LATG)	-15.200	5.051	0.006
	Motility disorder	23.300	13.142	0.089

Supplementary table 4. Surgical complication of each operator in totally laparoscopic total gastrectomy (TLTG) group.

	Operator A (n = 49)	Operator B (n = 118)	Operator C (n = 18)	Operator D (n = 38)	*P* value
Overall complication: n (%)	13 (26.5)	32 (27.1)	6 (33.3)	10 (26.3)	0.947
Major complication (≥ grade IIIa)	3 (6.1)	13 (11.0)	4 (22.2)	6 (15.8)	0.252
Comprehensive complication index	6.03 ± 11.33	7.27 ± 13.18	10.41 ±16.03	6.59 ± 12.63	0.664
Complication detected within 1 month					
Anastomotic complication					
Stenosis (EJ)	0 (0)	1 (0.8)	0 (0)	0 (0)	0.827
EJ leakage	0 (0)	4 (3.4)	1 (5.6)	1 (2.6)	0.544
JJ leakage	0 (0)	1 (0.8)	0 (0)	1 (2.6)	0.594
Duodenal stump leakage	1 (2.0)	0 (0)	0 (0)	1 (2.6)	0.356
Grade I	2 (4.1)	6 (5.1)	0 (0)	4 (10.5)	0.364
Grade II	8 (16.3)	19 (16.1)	3 (16.7)	3 (7.9)	0.629
Grade IIIa	3 (6.1)	11 (9.3)	4 (22.2)	6 (15.8)	0.186
Grade IIIb	0 (0)	1 (0.8)	1 (5.6)	0 (0)	0.159
Grade IVa	0 (0)	2 (1.7)	1 (5.6)	0 (0)	0.296
Grade IVb	0 (0)	1 (0.8)	0 (0)	0 (0)	0.827
Complication detected from 1 month to 1 year					
Delayed EJ stenosis (total no.)	3 (6.1)	9 (7.6)	0 (0)	2 (5.3)	0.651
Delayed EJ stenosis (≥grade IIIa)	3 (6.1)	2 (1.7)	0 (0)	2 (5.3)	0.335

Abbreviations: TLTG = Totally laparoscopic total gastrectomy; LATG = Laparoscopy-assisted total gastrectomy; EJ = esophagojejunostomy
